# Long-Lasting Desynchronization of Plastic Neuronal Networks by Double-Random Coordinated Reset Stimulation

**DOI:** 10.3389/fnetp.2022.864859

**Published:** 2022-04-19

**Authors:** Ali Khaledi-Nasab, Justus A. Kromer, Peter A. Tass

**Affiliations:** Department of Neurosurgery, Stanford University, Stanford, CA, United States

**Keywords:** coordinated reset stimulation, plastic neuronal networks, randomized stimulus amplitudes, long-lasting desynchronization, spike-timing-dependent plasticity

## Abstract

Hypersynchrony of neuronal activity is associated with several neurological disorders, including essential tremor and Parkinson’s disease (PD). Chronic high-frequency deep brain stimulation (HF DBS) is the standard of care for medically refractory PD. Symptoms may effectively be suppressed by HF DBS, but return shortly after cessation of stimulation. Coordinated reset (CR) stimulation is a theory-based stimulation technique that was designed to specifically counteract neuronal synchrony by desynchronization. During CR, phase-shifted stimuli are delivered to multiple neuronal subpopulations. Computational studies on CR stimulation of plastic neuronal networks revealed long-lasting desynchronization effects obtained by down-regulating abnormal synaptic connectivity. This way, networks are moved into attractors of stable desynchronized states such that stimulation-induced desynchronization persists after cessation of stimulation. Preclinical and clinical studies confirmed corresponding long-lasting therapeutic and desynchronizing effects in PD. As PD symptoms are associated with different pathological synchronous rhythms, stimulation-induced long-lasting desynchronization effects should favorably be robust to variations of the stimulation frequency. Recent computational studies suggested that this robustness can be improved by randomizing the timings of stimulus deliveries. We study the long-lasting effects of CR stimulation with randomized stimulus amplitudes and/or randomized stimulus timing in networks of leaky integrate-and-fire (LIF) neurons with spike-timing-dependent plasticity. Performing computer simulations and analytical calculations, we study long-lasting desynchronization effects of CR with and without randomization of stimulus amplitudes alone, randomization of stimulus times alone as well as the combination of both. Varying the CR stimulation frequency (with respect to the frequency of abnormal target rhythm) and the number of separately stimulated neuronal subpopulations, we reveal parameter regions and related mechanisms where the two qualitatively different randomization mechanisms improve the robustness of long-lasting desynchronization effects of CR. In particular, for clinically relevant parameter ranges double-random CR stimulation, i.e., CR stimulation with the specific combination of stimulus amplitude randomization and stimulus time randomization, may outperform regular CR stimulation with respect to long-lasting desynchronization. In addition, our results provide the first evidence that an effective reduction of the overall stimulation current by stimulus amplitude randomization may improve the frequency robustness of long-lasting therapeutic effects of brain stimulation.

## 1 Introduction

The human body can be viewed as a complex network with various interacting physiological systems. Stimulation of one system might have a strong and potentially even delayed impact on others (Bashan et al., 2012; Ivanov et al., 2016). A deeper understanding of the complex interactions between physiological systems through various signaling pathways and how they lead to the emergence of new physiological states might lead to the development of novel treatments for several diseases (Bartsch et al., 2015; Ivanov et al., 2016). For instance, a recent study on vibrotactile fingertip coordinated reset (CR) stimulation for the therapy of Parkinson’s disease (PD) revealed clinically and statistically significant improvement of motor control along with a significant decrease of abnormal, PD-related high-beta power in the sensorimotor cortex after 3 months of treatment (Pfeifer et al., 2021). Notably, sensory stimuli delivered to only a small part of the body were able to cause pronounced bilateral motor improvement of the entire body (Pfeifer et al., 2021).

PD patients typically suffer from pronounced motor symptoms, as well as several non-motor symptoms such as sensory impairments (Conte et al., 2013), impairments of sensorimotor integration (Abbruzzese and Berardelli, 2003), mood disorder, sleep disorder, and cognitive decline (Weiner et al., 2001; Reich et al., 2022). A network of strongly interconnected brain areas is involved, including the basal ganglia, the thalamus, and the sensorimotor cortex (McGregor and Nelson, 2019). Individual symptoms are associated with abnormally strong neuronal synchrony in these brain areas (Nini et al., 1995; Hammond et al., 2007).

An established treatment for medically refractory PD is high-frequency deep brain stimulation (HF DBS). Chronic delivery of HF DBS to target regions such as the subthalamic nucleus (STN) may suppress PD symptoms during stimulation (Krack et al., 2003; Krauss et al., 2021), where symptoms return shortly after cessation of stimulation (Temperli et al., 2003). Hence, permanent stimulation is required for persistent symptom relief, limiting battery life and, more importantly, increasing the risk of unwanted side effects, e.g., caused by stimulation of neighboring tissue due to current spread as well as stimulation of the very target region (Krack et al., 2002).

To substantially reduce the integral stimulation current, based on computational studies, it was suggested to develop DBS approaches that aim at specifically counteracting pathological synchrony by desynchronzation (Tass, 1999; Tass, 2000). Theory-based desynchronization techniques harness the nonlinear response of ensembles of coupled oscillators to external stimuli. For instance, an ensemble of synchronized oscillators can be desynchronized by delivering a single stimulus pulse at a vulnerable phase of the collective rhythm (Mines, 1914; Winfree, 1977; Warman and Durand, 1989; Tass, 1999). To reliably desynchronize an ensemble of coupled oscillators, it was suggested to deliver such a desynchronizing pulse shortly after a strong phase-resetting pulse (Tass, 2001; Tass, 2002; Zhai et al., 2005). A stimulation technique that does not rely on delivering stimuli at specific phases of the target rhythm is CR stimulation (Tass, 2003a). During CR stimulation, desynchronization is achieved by delivering phase-shifted stimuli to individual neuronal subpopulations. Other studies analyzed desynchronization by linear and nonlinear delayed feedback stimulation (Rosenblum M. and Pikovsky A., 2004; Rosenblum M. G. and Pikovsky A. S., 2004; Hauptmann et al., 2005a; Hauptmann et al., 2005b; Hauptmann et al., 2005c; Popovych et al., 2005; Popovych et al., 2006a; Popovych et al., 2006b; Pyragas et al., 2007; Popovych and Tass, 2010). Periodic stimulation at a suitable frequency may counteract neuronal synchrony by chaotic desynchronization (Wilson et al., 2011). Another study suggested closed-loop phasic burst stimulation during which burst stimuli are delivered at certain phases of the synchronous targeted rhythm (Holt et al., 2016). These phases were calculated based on online estimations of the phase response curve of the collective rhythm (Holt et al., 2016).

These stimulation techniques were originally developed for networks with fixed synaptic connections. The brain, however, is subject to synaptic plasticity and reorganizes network connectivity continuously (Liu et al., 2015; Van Ooyen and Butz-Ostendorf, 2017). One of these plasticity mechanisms is spike-timing-dependent plasticity (STDP), where the synaptic strengths are modulated according to the relative timings of post- and presynaptic spikes (Markram et al., 1997; Abbott and Nelson, 2000; Caporale and Dan, 2008). In many brain regions, STDP leads to a strengthening of synapses if the postsynaptic spike follows the presynaptic one and to a weakening of synapses if the presynaptic spike follows the postsynaptic one (Markram et al., 1997; Bi and Poo, 1998). STDP may enable the formation of neuronal assemblies (Litwin-Kumar and Doiron, 2014) and other network motifs (Ocker et al., 2015; Madadi Asl et al., 2018). It may also stabilize neuronal activity patterns, such as synchronous activity (Karbowski and Ermentrout, 2002), and lead to the coexistence of different stable states characterized by different activity patterns, such as desynchronized, synchronized, or cluster states (Seliger et al., 2002; Zanette and Mikhailov, 2004; Tass and Majtanik, 2006; Maistrenko et al., 2007; Masuda and Kori, 2007; Aoki and Aoyagi, 2009; Berner et al., 2020; Yanchuk et al., 2020).

Computational studies in plastic neuronal networks found that CR stimulation may induce not only acute desynchronization during stimulation but also long-lasting desynchronization that outlasts stimulation (Tass and Majtanik, 2006). The authors found that plastic synapses weakened during stimulation, which drove the network from a stable synchronous state, with strong synaptic connections, into the attractor of a stable desynchronized state, with weak synaptic connections. Based on these computational findings, CR stimulation was suggested as a possible therapy for inducing long-lasting therapeutic effects in movement disorders and epilepsies (Tass and Majtanik, 2006). In PD, corresponding long-lasting desynchronization and therapeutic effects were later confirmed by preclinical studies (Tass et al., 2009; Tass et al., 2012b; Wang et al., 2016; Wang et al., 2022) and clinical studies in PD patients delivering CR to the STN (Adamchic et al., 2014) or using vibrotactile fingertip CR stimulation (Tass, 2017; Syrkin-Nikolau et al., 2018; Pfeifer et al., 2021; Tass, 2022).

A deeper understanding of the parameter dependence of long-lasting desynchronization effects is critical for future clinical applications. In an earlier computational study, the long-lasting desynchronization effects of CR stimulation were studied in networks of Hodgkin-Huxley neurons with STDP (Manos et al., 2018). CR stimulation with two spatio-temporal stimulus patterns was delivered: CR with rapidly varying sequence (CR RVS) and CR with slowly varying sequence (CR SVS). For CR RVS the stimulus pattern is varied after each subpopulation received one stimulus, whereas the pattern remains fixed for longer time intervals during CR SVS. The authors reported that long-lasting desynchronization effects of strong CR RVS and CR SVS stimulation were sensitive to the ratio between the stimulation frequency and the frequency of the pathological synchronous rhythm. In contrast, for weak stimulation amplitudes, long-lasting desynchronization effects became more robust with respect to changes of the stimulation frequency, especially for CR RVS, whereas CR SVS did not induce long-lasting desynchronization for certain unfavorable frequency ratios. A similar observation was made in a later computational study analyzing long-lasting effects of CR RVS stimulation in networks of leaky integrate-and-fire (LIF) neurons with STDP (Kromer and Tass, 2020). There, it was hypothesized that certain unfavorable stimulation frequencies might lead to resonances and stabilize synchronous neuronal activity. Another computational study using networks of LIF neurons with STDP found that the stimulation frequency also needs to be adjusted to the time scales of synaptic long-term depression (LTD) and long-term potentiation (LTP) (Kromer et al., 2020). From a theoretical standpoint, these frequency dependences might limit clinical applicability as different PD symptoms have been associated with excessive neuronal synchrony in different frequency bands. Specifically, dyskinesia and tremor have been associated with synchronous neuronal activity in the theta band (3–10 Hz) (Brown, 2003; Steigerwald et al., 2008; Tass et al., 2010; Contarino et al., 2012). In contrast, rigidity and bradykinesia have been associated with synchronized beta-band activity (13–30 Hz) (Kühn et al., 2006; Weinberger et al., 2006). Furthermore, multiple central oscillators were found to underlie the generation of tremor (Raethjen et al., 2000).

To improve the robustness of long-lasting desynchronization effects with respect to changes of the stimulation frequency, recent computational studies suggested temporal randomization of the CR stimulus pattern. Typically CR stimulation is delivered with a fixed cycle period, such that each stimulation site is activated exactly once per cycle (Tass, 2003a). A recent series of computational and theoretical studies found that the frequency robustness of long-lasting desynchronization effects increased if stimulus delivery times were randomized. In particular, stimulus deliveries according to a Poisson pulse train (Kromer and Tass, 2020; Khaledi-Nasab et al., 2021a) and the addition of random jitters to the individual stimulus delivery times within individual CR cycles were considered (Khaledi-Nasab et al., 2021b).

In the present study, we suggest CR stimulation with randomized stimulus amplitudes to increase the robustness of long-lasting desynchronization effects of CR stimulation with respect to the stimulation frequency. We perform computer simulations of networks of LIF neurons with STDP and study long-lasting effects of regular CR (Popovych and Tass, 2012; Zeitler and Tass, 2015) and temporally uncorrelated CR (referred to as uncorrelated multichannel noisy stimulation in Zeitler and Tass (2018)). Based on results from previous studies, we expect long-lasting effects of regular CR to show a pronounced frequency dependence (Manos et al., 2018; Kromer et al., 2020) and long-lasting effects of temporally uncorrelated CR to be more robust with respect to changes of the stimulation frequency (Kromer et al., 2020; Kromer and Tass, 2020; Khaledi-Nasab et al., 2021a; Khaledi-Nasab et al., 2021b). We compare long-lasting desynchronization effects for both patterns with and without randomized stimulus amplitudes. Remarkably, double-random CR stimulation, i.e., CR stimulation with randomization of stimulus amplitudes as well as temporally uncorrelated stimulus times, substantially improves the long-term desynchronization outcome for high CR stimulation frequencies (compared to the intrinsic frequency of the abnormal target rhythm) and large numbers of separately stimulated subpopulations.

This present paper is organized as follows: In section 2, we present details on the plastic neuronal network model used throughout the paper. Then, the different stimulation patterns and stimulus randomizations are introduced. We also present the detailed derivation of our theoretical approximations for the synaptic weight dynamics during ongoing stimulation. In section 3, we compare results for CR stimulation patterns with constant stimulus amplitudes and with randomized stimulus amplitudes. In particular, we consider uniformly distributed stimulus amplitudes and binarily distributed stimulus amplitudes. For the latter, a random fraction of stimuli is removed from the pattern (by setting its amplitudes to zero) while the remaining stimuli possess a constant amplitude. Finally, in section 4, we discuss our results in the context of the current literature and point out possible consequences for clinical studies.

## 2 Model and Method

Simulations were performed for networks of *N* = 10^3^ excitatory LIF neurons with STDP. All parameters were chosen according to Kromer et al. (2020), Khaledi-Nasab et al. (2021a), and Khaledi-Nasab et al. (2021b) such that a stable desynchronized state, with weak synaptic connections, and a stable synchronized state, with strong synaptic connections, coexist. All equations and parameters are given in Kromer et al. (2020), Khaledi-Nasab et al. (2021a), and Khaledi-Nasab et al. (2021b) and are presented in Supplementary Material for the reader’s convenience.

### 2.1 Network Connectivity

We considered a spatial network of LIF neurons with distance-dependent connection probability. The LIF neurons were equidistantly spaced in the interval *x*_*i*_ ∈ [0, *L*]. *L* sets the length scale over which neurons are distributed in space. The probability for a synaptic connection between neurons was distance dependent and proportional to exp((|*x*_*j*_ − *x*_*i*_|)/0.1*L*) (Ebert et al., 2014). Synapses were implemented such that each neuron had 0.07*N* outgoing synapses. If not stated otherwise, simulation results were averaged over three network realizations that differed in the realization of random synaptic connectivity, of initial synaptic weights, and neuron parameters (Supplementary Material).

We considered a fixed synaptic transmission delay of *τ* = 3 ms (Kromer et al., 2020).

### 2.2 Spike-Timing-Dependent Plasticity

The dynamics of synaptic weights, *w*_*ij*_(*t*), was determined by STDP. We considered a nearest-neighbor STDP scheme in which weight updates are performed at postsynaptic spike times and presynaptic spike arrival times (Morrison et al., 2008). Corresponding weight updates, *w*_*ij*_ → *w*_*ij*_ + *W*(*t*_*j*_ − (*t*_*i*_ + *t*_d_)), are given by the STDP function (Song et al., 2000; Kromer and Tass, 2020)

$$W\left(\Delta t\right)=\eta \left\{\begin{array}{cc} \;e^{-|\Delta t|/\tau_{+}},\; & \;\Delta t>0\\ 0,\; & \;\Delta t=0\\ -\frac{\beta }{\tau_{R}}\;e^{-|\Delta t|/\tau_{-}},\; & \;\Delta t<0 \end{array}\right..$$ (1)

Δ*t* = *t*_*j*_ − (*t*_*i*_ + *t*_d_) is the time lag between the postsynaptic spike time, *t*_*j*_, and the latest presynaptic spike arrival time, *t*_*i*_ + *t*_d_, (when the update is triggered by a postsynaptic spike), and the time lag between the presynaptic spike arrival time, *t*_*i*_ + *t*_d_, and the latest postsynaptic spike time, *t*_*j*_, (when the update is triggered by a presynaptic spike arrival). *η* = 0.02 scales the weight update per spike, *τ*_R_ = 4 yields asymmetry in STDP decay times *τ*_+_ = 10 ms and *τ*_-_ = *τ*_+_ *τ*_R_. *β* = 1.4 scales the ratio of overall LTD to LTP. In addition to this dynamics, individual synaptic weights are restricted to the interval *w*_*ij*_ ∈ [0, 1] by hard bounds (Song et al., 2000; Rubin et al., 2001).

For these parameters a stable synchronized state and a stable desynchronized state coexist, see Kromer and Tass (2020); Kromer et al. (2020) and Khaledi-Nasab et al. (2021a) for details. In these states, individual synaptic weights approach the hard bounds. The mean synaptic weight in the desynchronized state approaches small values as most individual synaptic weights approach the lower hard bound. In contrast, the value of the mean synaptic weight in the synchronized state is approximately 0.38, indicating that about 38% of the synapses approach the upper hard bound. Snapshots of the distributions of the individual synaptic weights in a similar network model in either state can be found in Figures 8C,D in Kromer and Tass (2020).

### 2.3 Stimulation

In the present paper, we studied the network’s response to randomized spatio-temporal stimulus patterns. Individual stimuli were modeled as charge-balanced electrical pulses, consisting of an excitatory rectangular pulse of duration *ν*_e_ = 0.5 ms and an inhibitory rectangular pulses of duration *ν*_i_ = 3 ms. This asymmetry was motivated by preclinical and clinical studies on CR stimulation (Tass et al., 2012b; Adamchic et al., 2014; Wang et al., 2016). The stimulation amplitude was scaled by the dimensionless parameter *A*_stim_ such that the excitatory pulse had the amplitude *A*_stim_ *μ*/*ν*_e_ and the inhibitory one the amplitude − *A*_stim_ *μ*/*ν*_i_ with *μ* = (*V*_th__,__spike_ − *V*_reset_)/⟨*C*_*i*_⟩ (see appendix for more details). Throughout the present paper, we considered different stimulus patterns that were motivated by the original CR stimulation pattern (Tass, 2003a; Tass, 2003b) and recently studied randomized versions of this pattern (Zeitler and Tass, 2018; Khaledi-Nasab et al., 2021b). In particular, we applied CR stimulation with randomized stimulation times (tCR), studied in a previous computational study (Zeitler and Tass, 2018) (there it was called uncorrelated multichannel noisy stimulation), and CR stimulation with randomized stimulation amplitudes (CR). Following, we present a detailed description of all stimulation patterns considered throughout the present paper.

**FIGURE 1:**
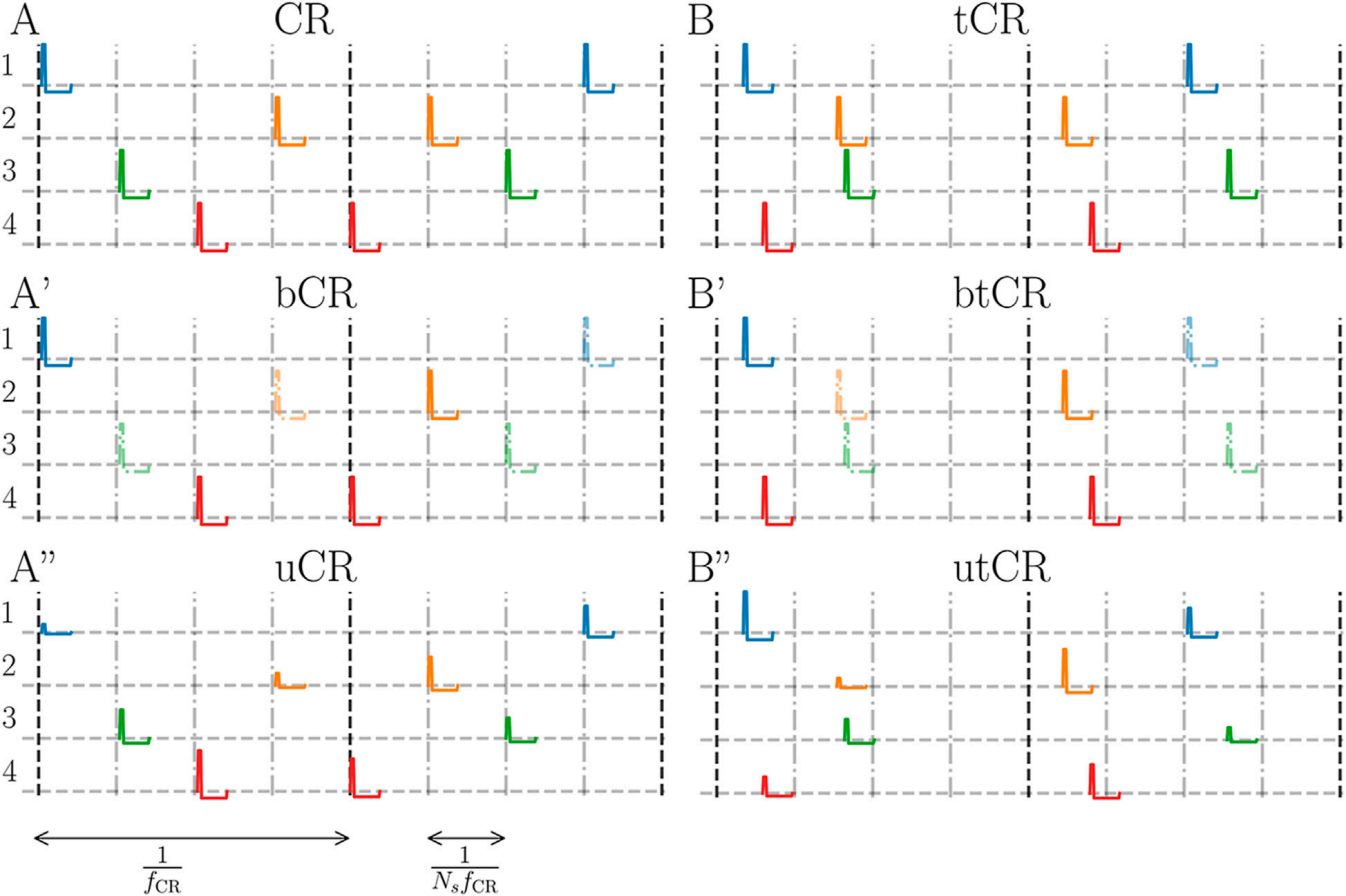
Illustration of stimulation patterns. **(A)**: Regular CR stimulation for *N*_*s*_ = 4 subpopulations where the stimuli are delivered at integer multiples of 1/*N*_*s*_ *f*_CR_ (vertical dashed-dotted gray lines) with fixed stimulus amplitude *A*_stim_ = 1. The vertical black dashed lines show the beginnings of new stimulation cycles. Colors indicate stimuli delivered to the same subpopulation (rows). A’: A representative bCR stimulation pattern where only a fraction of *p* = 0.5 of the stimuli is delivered. Stimuli that were removed (*A*_stim_ = 0) are plotted translucently. A”: A uCR stimulation pattern. Stimulus amplitudes are uniformly distributed between *A*_stim_ = 0 and *A*_stim_ = 1 (see colored stimuli in panel A″). **(B)**: tCR stimulation where stimulus times are uniformly distributed within CR cycles. Panels B′ and B″ show the btCR and utCR stimulation patterns with binarily distributed (B′) and uniformly distributed (B″) stimulus amplitudes, respectively. These patterns possess the same statistics of stimulus delivery times as the tCR pattern; however, for the btCR pattern only a fraction *p* = 0.5 of stimuli is delivered (removed stimuli are plotted translucently). For the utCR pattern, stimulus amplitudes are uniformly distributed in the interval *A*_stim_ ∈ [0, 1].

• **Coordinated reset (CR) stimulation:** The CR stimulation pattern is characterized by the stimulation frequency, *f*_CR_, which sets the CR cycle period, 1/*f*_CR_, and the number of separately stimulated subpopulations, *N*_s_. During each CR cycle, each subpopulation receives exactly one stimulus. Obeying this restriction, individual stimuli are delivered at subsequent multiples of 1/*N*_s_ *f*_CR_ to random subpopulations. A representative realization of a CR pattern is illustrated in Figure 1A. The CR pattern was previously introduced as CR with rapidly varying sequence (Popovych and Tass, 2012; Zeitler and Tass, 2015) and was used in preclinical and clinical studies (Tass et al., 2012b; Adamchic et al., 2014; Wang et al., 2016). Here, we will use the term “regular CR” to emphasize the difference to the different randomized CR patterns used throughout the paper and introduced in the following.
• **Temporally uncorrelated CR (tCR):** Same as CR stimulation, however, each subpopulation receives exactly one stimulus per cycle at a uniformly distributed time between zero and 1/*f*_CR_. There is no correlation between the stimulus times of different channels. A representative realization of a tCR pattern is illustrated in Figure 1B. The tCR pattern was originally introduced in Zeitler and Tass (2018), where it was referred to as uncorrelated multichannel noisy stimulation.
• **CR with binarily (bCR) or uniformly distributed (uCR) stimulus amplitudes:** Same as regular CR stimulation but the amplitude of each stimulus is randomly chosen according to either a uniform (uCR) and a binary distribution (bCR). For the former, stimulus amplitudes were uniformly distributed between *A*_stim_ = 0 and *A*_stim_ = 1. Corresponding stimulus patterns will be marked by a lower case ‘u’, e.g., uCR for regular CR with uniformly distributed stimulus amplitudes. One representative realization of a uCR pattern is shown in Figure 1A. For binarily distributed stimulus amplitudes, stimuli possess either the amplitude *A*_stim_ = 1 or *A*_stim_ = 0. This corresponds to the random removal of a fraction 1 − *p* of stimuli from the pattern. *p* will be referred to as *fraction of delivered stimuli* in the following. The probability *p* at which stimuli have amplitude *A*_stim_ = 1 is a free parameter. A lower case ‘b’ will mark corresponding stimulus patterns, e.g., bCR for regular CR with binarily distributed stimulus amplitudes.
• **Double random CR stimulation:** Same as tCR stimulation except that, in addition, stimulus amplitudes were randomized using either one of the distributions introduced above. Double random CR stimulation combines randomized stimulus times and randomized stimulus amplitudes. Double random CR with uniformly distributed stimulus amplitudes will be referred to as utCR stimulation and double random CR with binarily distributed stimulus amplitudes as btCR stimulation. Representative realizations of utCR and btCR stimulation patterns are shown in Figures 1B,B′, respectively.

### 2.4 Measures of Synchronization and Data Evaluation

To measure the degree of neuronal synchrony, we calculated the time-averaged Kuramoto order parameter (Kuramoto, 1984) over *T*-seconds intervals as

$$\bar{\rho }\left(t\right)=\frac{1}{T}\overset{t+\frac{T}{2}}{\underset{t-\frac{T}{2}}{\int }}dt^{\prime }\left|\frac{1}{N}\overset{N-1}{\underset{k=0}{\sum }}e^{2\pi I\psi_{k}\left(t^{\prime }\right)}\right|.$$ (2)

*N* is the number of neurons, *T* = 10 s the averaging time interval, and *ψ*_*k*_(*t*) is a phase function associated with the interspike intervals of neuron *k*. *ψ*_*k*_(*t*) attains subsequent integer values at the spike times of neuron *k* and increases linearly in time during interspike intervals (Rosenblum et al., 2001). Thus, $\bar{\rho }\left(t\right)$ measures the degree of in-phase synchronized spiking on the time scale *T* with $\bar{\rho }\approx 0$ indicating the lack of in-phase synchronized spiking and $\bar{\rho }=1$ indicating perfect in-phase synchronized spiking.

The focus of our analysis was to distinguish between long-lasting desynchronization and long-lasting synchronization of neuronal spiking activity. For this purpose, a quantification of the degree of neuronal synchrony with the Kuramoto order parameter, Eq. 2, is sufficient. During stimulation, more complex spike patterns that require a more detailed analysis, e.g., cluster states, may occur, see, for instance, Figure 3 in Kromer and Tass (2020).

### 2.5 Preparation of Networks in a Stable Synchronized State

Prior to stimulation, networks were prepared in a stable synchronized state, which was studied in detail in Kromer et al. (2020). The preparation was done by initializing the weights of all synapses randomly such that an initial mean synaptic weight of ⟨*w*⟩ = 0.8 was realized. We simulated the networks for 500 s until the mean synaptic weight approached a stationary value of ⟨*w*⟩ ≈ 0.38 corresponding to a stable synchronized state (Kromer et al., 2020). In this state, the Kuramoto order parameter, Eq. 2, was close to one, indicating a strong degree of in-phase synchronization of neuronal spiking. Synchronized neuronal spiking events occurred at a frequency of ≈3.5 Hz, which will be considered as the frequency of the synchronous rhythm that is targeted by the stimulation.

### 2.6 Quantification of Acute Effects, Acute After-Effects, and Long-Lasting After-Effects of Stimulation

In the present paper, we focus on the effect of stimulation on neuronal synchrony. To quantify this effect, we evaluated the Kuramoto order parameter (Eq. (2)). We distinguish between *acute effects*, observable during stimulation; *acute after-effects*, observable shortly after cessation of stimulation; and long-lasting after-effects, briefly denoted as *long-lasting effects*, observable in the long-time limit after cessation of stimulation when the neuronal network has relaxed to a stable state. The acute effect of stimulation was quantified by time-averaging the Kuramoto order parameter ${\bar{\rho }}_{\text{ac}}$ over a 10 seconds time interval at the end of the stimulation duration. In addition, we recorded the mean synaptic weight ⟨*w*⟩_ac_ at the end of the stimulation duration to quantify the effect of stimulation on the synaptic connectivity. The acute after-effect of stimulation was quantified by time-averaging the Kuramoto order parameter ${\bar{\rho }}_{\text{af}}$ over the first 10 seconds time interval after cessation of stimulation. Finally, long-lasting effects were quantified by time-averaging the Kuramoto order parameter ${\bar{\rho }}_{\text{ll}}$ over a 10 seconds time interval 1,000 s after cessation of stimulation.

### 2.7 Estimated Mean Rate of Synaptic Weight Change

Previous studies presented theoretical approximations of the mean rate of weight change during ongoing stimulation (Kromer and Tass, 2020). In particular, results for CR stimulation (Kromer et al., 2020) and randomized versions of CR stimulation (Khaledi-Nasab et al., 2021b) were derived for the limit of *stimulation-controlled spiking*. In this limit, each spike is triggered by a stimulus and each stimulus causes a spike; thus, neuronal spiking due to the intrinsic dynamics or any input other than the stimulation, e.g., noise or synaptic input, is neglected. We further assume that stimuli trigger spikes at a fixed time lag, *Δ*, i.e., variability of the time delay between stimulus delivery and the triggered neuronal spike can be neglected. This may be a valid assumption, for instance, for strong direct stimulation of the neuronal Soma and for antidromic stimulation of cortical neurons during STN DBS. For the latter, high response fidelity was found by experimental studies (Li et al., 2007). In the following, we set Δ = 0. However, our results also apply to any constant nonzero Δ. Previous studies on networks of LIF neurons using the nearest-neighbor STDP scheme (Eq. 1) found that these assumptions are valid for strong (*A*_stim_ ≈ 1) and fast stimulation, i.e., if the stimulation frequency is fast compared to that of the synchronous target rhythm (Kromer et al., 2020; Kromer and Tass, 2020; Khaledi-Nasab et al., 2021b). Then, the mean rate of weight change for individual synapses results from the statistics of time lags between subsequent stimuli to the post- and presynaptic neuron, respectively (Kromer and Tass, 2020; Khaledi-Nasab et al., 2021b).

We apply the same approach to derive theoretical approximations for the mean rate of weight change of synapses during ongoing CR stimulation with randomized stimulus patterns btCR and bCR for arbitrary fractions of delivered stimuli *p*. Note that the CR and tCR patterns are covered by the results for bCR and btCR patterns in the limit of *p* = 1, respectively.

**FIGURE 2:**
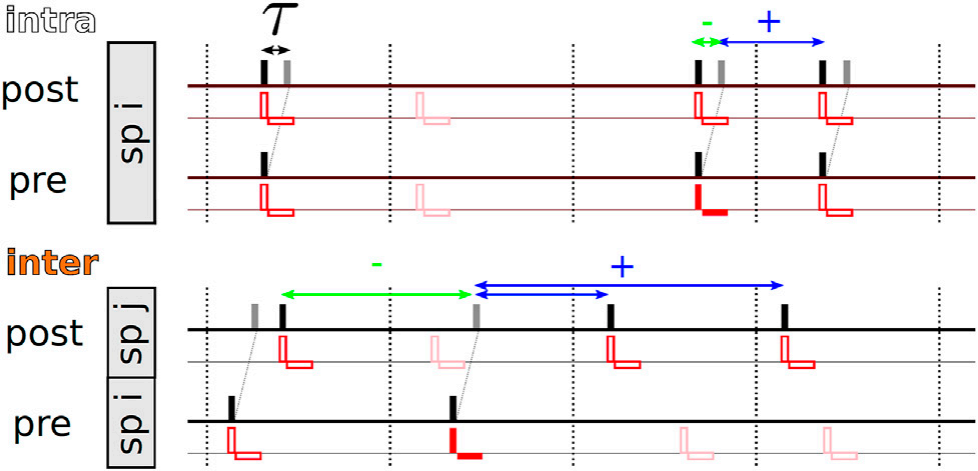
Illustration of the stimulation-controlled spiking approximation for tCR stimulation and time lags contributing to weight updates according to the nearest-neighbor STDP scheme. For the derivation of the theoretical approximation for the mean rate of weight change, Eq. 4, we assume stimulation-controlled spiking, i.e., each spike (black bars) is caused by a stimulus (red vertical and horizontal bars, illustrating biphasic pulses) and each stimulus triggers a spike in neurons in the corresponding subpopulation (sp). In the upper row, we show representative spike trains for pre- and postsynaptic neurons for an intrapopulation synapse, i.e., both neurons are part of subpopulation *i*. According to the nearest-neighbor STDP scheme, Eq. 1, synaptic weight updates are triggered by two events: First, when a presynaptic spike arrives at the postsynaptic neuron (gray bar), weight updates are based on negative time lags between the presynaptic spike arrival and the latest postsynaptic spike (green double-headed arrow). Second, when a postsynaptic spike occurs, weight updates are based on positive time lags resulting from pairings of the postsynaptic spike with the latest presynaptic spike arrival (blue double-headed arrow). All time lags that involve the arrival time of the presynaptic spike triggered by the red-marked stimulus are marked by horizontal double-headed arrows. For tCR stimulation, a fraction of stimuli is removed from the stimulus pattern and does not trigger spikes. This is illustrated by plotting removed stimuli translucently. The bottom row shows representative pre- and postsynaptic spike trains for an interpopulation synapse (inter), i.e., post- and presynaptic neurons are part of different subpopulations. Here, post- and presynaptic neurons typically receive stimuli at different times. Again, we illustrate all time lags involving pairings with the arrival time of the presynaptic spike triggered by the red-marked stimulus by horizontal arrows. Note that multiple postsynaptic spikes may be paired with the same presynaptic spike arrival if no further presynaptic spikes arrive between them (see the two blue horizontal arrows). Vice versa, multiple presynaptic spike arrivals might be paired with the same postsynaptic spike if no postsynaptic spikes occur between them.

For a given stimulation pattern X, the statistics of time lags between subsequent stimuli delivered to the post- and the presynaptic neuron, respectively, depends on the considered type of synapse. Following previous studies (Kromer and Tass, 2020), we distinguish between *intra-* and *interpopulation* synapses. Intrapopulation synapses connect neurons in the same subpopulation, whereas interpopulation synapses connect neurons in different subpopulations (Kromer and Tass, 2020). A corresponding schematic is shown in Figure 2.

The mean rate of weight change is solely determined by the statistics of time lags, *s*, between stimulus deliveries to the presynaptic and postsynaptic neuron

$$p_{\text{intra/inter}}^{\text{X}}\left(s\right)=p_{\text{intra/inter}}^{+,\text{X}}\left(s\right)+p_{\text{intra/inter}}^{-,\text{X}}\left(s\right).$$ (3)

$p_{\text{intra/inter}}^{+,\text{X}}\left(s\right)$ and $p_{\text{intra/inter}}^{-,\text{X}}\left(s\right)$ are the distributions of time lags, *s*, that lead to positive (+) and negative (-) weight updates for intra- and interpopulation synapses, respectively, for the considered nearest-neighbor STDP scheme, see above. Negative weight updates result from pairings of delayed presynaptic spike arrivals with postsynaptic spikes triggered by the same (intrapopulation synapses) or the latest stimulus delivered to the subpopulation of the postsynaptic neuron (interpopulation synapses). Positive weight updates result from pairings of postsynaptic spikes with the latest delayed presynaptic spike arrivals caused by the latest stimulus delivered to the presynaptic neuron, see Figure 2.

An estimate of the mean rate of weight change is given by (Kromer and Tass, 2020)

$$J_{\text{intra/inter}}^{\text{X}}=pf_{\text{CR}}\int ds\;p_{\text{intra/inter}}^{\text{X}}\left(s\right)W\left(s-\tau \right).$$ (4)

*W*(*s*) is the STDP function, Eq. 1, and *τ* the synaptic delay time. The prefactor *pf*_CR_ is the average number of spikes per second of a single neuron during ongoing stimulation. On average, each spike is paired with two other spikes, the latest and the next spike of the presynaptic/postsynaptic neuron; thus, the integral over $p_{\text{intra/inter}}^{\text{X}}\left(s\right)$ yields a value of two. This is illustrated in Figure 2.

Following, we derive expressions for the distributions of interstimulus intervals leading to positive weight updates $\left(p_{\text{intra/inter}}^{+,\text{X}}\left(s\right)\right)$ and to negative $\left(p_{\text{intra/inter}}^{-,\text{X}}\left(s\right)\right)$ weight updates for both btCR and bCR stimulation.

#### 2.7.1 Temporally Uncorrelated CR With Binary Amplitude Randomization

For btCR stimulation, individual stimuli are delivered at uniformly distributed times during CR cycles of period 1/*f*_CR_. The resulting distribution of interstimulus intervals between two stimuli is given by

$$q\left(s,s_{0}\right)=f_{\text{CR}}^{2}\left\{\begin{array}{cc} \frac{1}{f_{\text{CR}}}-|s-s_{0}|,\; & |s-s_{0}|\leq \frac{1}{f_{\text{CR}}}\\ 0,\; & \text{otherwise} \end{array}\right..$$ (5)

Here, *s*_0_ is the mean interstimulus interval. *s*_0_ = *k*/*f*_CR_, if the postsynaptic neuron receives the stimulus *k* CR cycles after (*k* > 0) or before (*k* < 0) the presynaptic neuron, or if both neurons receive stimuli in the same CR cycle (*k* = 0).

We introduce the probabilities

$$P_{\text{nbd}}=\overset{\tau }{\underset{0}{\int }}ds^{\prime }q\left(s^{\prime },\frac{1}{f_{\text{CR}}}\right)$$ (6)

that the next stimulus is delivered to the postsynaptic neuron before the delayed presynaptic spike arrives, i.e., after the delay time *τ*; and the probability

$$P_{\text{cbd}}=\overset{\tau }{\underset{-\frac{1}{f_{\text{CR}}}}{\int }}ds^{\prime }q\left(s^{\prime },0\right)$$ (7)

that during the current CR cycle the stimulus delivered to the postsynaptic neuron is delivered before the presynaptic spike arrives at the postsynaptic neuron. Here, the subscripts “nbd” and “cbd” stand for “next before delay” and “current before delay”, respectively.

For intrapopulation synapses, positive weight updates only result from time lags *s* > *τ*. Such time lags may result from pairings of delayed presynaptic spike arrivals with postsynaptic spikes triggered by subsequent stimuli. For the distribution of corresponding interstimulus intervals, we find

$$\begin{array}{ccc} p_{\text{intra}}^{+,\text{btCR}}\left(s\right)=p\left[q\left(s,\frac{1}{f_{\text{CR}}}\right)+pP_{\text{nbd}}\overset{\infty }{\underset{m=2}{\sum }}{\left(1-p\right)}^{m-2}q\left(s,\frac{m}{f_{\text{CR}}}\right)\right] & & \\ +\left(1-p\right)p\overset{\infty }{\underset{m=2}{\sum }}{\left(1-p\right)}^{m-2}q\left(s,\frac{m}{f_{\text{CR}}}\right),\;\;s\geq \tau . & & \end{array}$$ (8)

Negative weight updates result from time lags *s* < *τ*. Thus, we set $p_{\text{intra}}^{+,\text{btCR}}\left(s\right)=0$ for *s* < *τ*. Negative time lags result from pairings of delayed presynaptic spike arrivals with the latest postsynaptic spike. The distribution of corresponding interstimulus intervals is given by

$$p_{\text{intra}}^{-,\text{btCR}}\left(s\right)=p\left[q\left(s,\frac{1}{f_{\text{CR}}}\right)+\left(1-P_{\text{nbd}}\right)\delta \left(s\right)\right]+\left(1-p\right)\delta \left(s\right),\;\;s<\tau .$$ (9)

Accordingly, we set $p_{\text{intra}}^{-,\text{btCR}}\left(s\right)=0$ for *s* ≥ *τ*. The first and second terms account for the cases where a stimulus occurs in the next CR cycle and no stimulus occurs in the next CR cycle, respectively. Here, *δ*(*s*) is the Dirac delta distribution.

For interpopulation synapses, positive weight updates result from pairings of delayed presynaptic spike arrivals with postsynaptic spikes triggered by stimuli that either occur in the same cycle (but after the presynaptic spike arrives at the postsynaptic neuron) or by subsequent stimuli. We find

$$\begin{array}{ccc} p_{\text{inter}}^{+,\text{btCR}}\left(s\right) & = & p(q\left(s,0\right)+P_{\text{cbd}}[p\left(q\left(s,\frac{1}{f_{\text{CR}}}\right)+pP_{\text{nbd}}\overset{\infty }{\underset{m=2}{\sum }}{\left(1-p\right)}^{m-2}q\left(s,\frac{m}{f_{\text{CR}}}\right)\right)\\ & & +\left.\left.\left(1-p\right)p\overset{\infty }{\underset{m=2}{\sum }}{\left(1-p\right)}^{m-2}q\left(s,\frac{m}{f_{\text{CR}}}\right)\right]\right)\\ & & +\left(1-p\right)p\left(q\left(s,\frac{1}{f_{\text{CR}}}\right)+pP_{\text{nbd}}\overset{\infty }{\underset{m=2}{\sum }}{\left(1-p\right)}^{m-2}q\left(s,\frac{m}{f_{\text{CR}}}\right)\right)\\ & & +p\overset{\infty }{\underset{m=2}{\sum }}{\left(1-p\right)}^{m}q\left(s,\frac{m}{f_{\text{CR}}}\right),\;\;s\geq \tau . \end{array}$$ (10)

We set $p_{\text{inter}}^{+,\text{btCR}}\left(s\right)=0$ for *s* < *τ*. The first three rows account for the case where the postsynaptic neuron receives a stimulus in the same CR cycle as the presynaptic neuron, and considers the probabilities for parings of the presynaptic spike arrival with the postsynaptic spike resulting from that stimulus or with postsynaptic spikes resulting from subsequent stimuli. The term in the fourth row accounts for the case where the postsynaptic neuron does not receive a stimulus during the same cycle as the presynaptic neuron. Thus, the presynaptic spike arrival is paired with postsynaptic spikes resulting from subsequent stimuli. The term in the last row considers the case where the postsynaptic neuron neither receives a stimulus in the current nor in the next cycle.

Negative time lags may result from pairings of presynaptic spike arrivals with the latest postsynaptic spike. For the corresponding distribution of interstimulus intervals, we find

$$\begin{array}{ccc} p_{\text{inter}}^{-,\text{btCR}}\left(s\right) & = & p(q\left(s,\frac{1}{f_{\text{CR}}}\right)+\left(1-P_{\text{nbd}}\right)[\\ & & p\left(q\left(s,0\right)+\left(1-P_{\text{cbd}}\right)p\overset{\infty }{\underset{m=1}{\sum }}{\left(1-p\right)}^{m-1}q\left(s,-\frac{m}{f_{\text{CR}}}\right)\right)\\ & & \left.\left.+\left(1-p\right)p\overset{\infty }{\underset{m=1}{\sum }}{\left(1-p\right)}^{m-1}q\left(s,-\frac{m}{f_{\text{CR}}}\right)\right]\right)\\ & & +\left(1-p\right)\left(p\left(q\left(s,0\right)+\left(1-P_{\text{cbd}}\right)p\overset{\infty }{\underset{m=1}{\sum }}{\left(1-p\right)}^{m-1}q\left(s,-\frac{m}{f_{\text{CR}}}\right)\right)\right.\\ & & +\left.\left(1-p\right)p\overset{\infty }{\underset{m=1}{\sum }}{\left(1-p\right)}^{m-1}q\left(s,-\frac{m}{f_{\text{CR}}}\right)\right),\;\;s<\tau . \end{array}$$ (11)

We set $p_{\text{inter}}^{-}\left(s\right)=0$ for *s* ≥ *τ*. Here, the first three rows account for the case where the postsynaptic neuron receives a stimulus one CR cycle after the presynaptic neuron, and the last two rows account for the case where it does not receive a stimulus in that cycle.

#### 2.7.2 CR With Binary Amplitude Randomization

Next, we consider the expected mean rate of weight change for bCR stimulation. Results for *p* = 1 were derived in Kromer et al. (2020). There, using the stimulation-controlled spiking approximation, results for the case *f*_CR_ < 1/*N*_s_ *τ* were derived, i.e., when presynaptic spikes arrive at the postsynaptic neuron before the next stimulus is delivered. To incorporate stimulation with rather high stimulation frequencies (1/*N*_s_ *τ* < *f*_CR_ < 2/*N*_s_ *τ*), the authors derived correction terms $\delta p_{\text{intra/inter}}^{\text{X}}\left(s\right)$ . Following, we consider the case of *p* ≤ 1. We follow a similar approach as in Kromer et al. (2020) and derive results for $p_{\text{intra/inter}}^{\text{X}}\left(s\right)$ and the correction terms $\delta p_{\text{intra/inter}}^{\text{X}}\left(s\right)$ . Similar to the derivation for btCR, we assume that neurons spike instantaneously when they receive a stimulus.

We first consider the case of stimulation with stimulation frequencies that fulfill *f*_CR_ < 1/*N*_s_ *τ*. Corresponding results are marked by the index “0”. For such *f*_CR_, presynaptic spikes arrive at the postsynaptic neuron before the next stimulus is delivered. We derive results for $p_{\text{intra/inter},0}^{\text{bCR}}\left(s\right)$ by considering all possible CR patterns and the interstimulus intervals that lead to positive and negative weight updates.

For intrapopulation synapses, negative weight updates can only result from pairings of presynaptic spike arrivals with postsynaptic spikes that were caused by the same stimulus,

$$p_{\text{intra},0}^{-,\text{bCR}}\left(s\right)=\delta \left(s\right).$$ (12)

Positive weight updates may result from pairings of presynaptic spike arrivals with the postsynaptic spikes resulting from the next stimulus delivered to the postsynaptic neuron. For *p* = 1, this stimulus always occurs in the next CR cycle. The corresponding $p_{\text{intra},0}^{+,\text{CR}}\left(s\right)$ was derived in Kromer et al. (2020). For *p* ≤ 1, the next stimulus occurs after *k* cycles with probability *p*(1 − *p*)^*k*^. This yields,

$$p_{\text{intra},0}^{+,\text{bCR}}\left(s\right)=p\overset{\infty }{\underset{k=0}{\sum }}{\left(1-p\right)}^{k}\overset{2N_{\text{s}}}{\underset{m=1}{\sum }}\frac{N_{\text{s}}-|m-N_{\text{s}}|}{N_{\text{s}}^{2}}\delta \left(s-\frac{m+N_{\text{s}}k}{N_{\text{s}}f_{\text{CR}}}\right),\;\;s\geq \tau .$$ (13)

For *s* < *τ*, we set $p_{\text{intra},0}^{+,\text{bCR}}\left(s\right)$ to zero.

For interpopulation synapses, the presynaptic and postsynaptic neurons receive stimuli at different times that are multiples of 1/*N*_s_ *f*_CR_. Results for *p* = 1 were derived in Kromer et al. (2020). For *p* ≤ 1, given a presynaptic stimulus, the postsynaptic neuron receives a stimulus in the same CR cycle as the presynaptic one with probability *p*. The latter stimulus can occur either before or after the presynaptic spike arrival and, thus, a pairing between these spikes either results in a negative or positive weight update. The weight update with the opposite sign occurs between the presynaptic spike arrival and a postsynaptic spike triggered by a stimulus in a later CR cycle (for a positive weight update) or in a previous cycle (for a negative weight update), respectively. This stimulus occurs with probability *p*(1 − *p*)^*k*^, *k* cycles after/before the current CR cycle. For the resulting terms for $p_{\text{inter},0}^{\pm ,\text{bCR}}\left(s\right)$ , we find

$$\begin{array}{ccc} p_{\text{inter},0}^{\pm ,\text{bCR}}\left(s\right) & = & \frac{p}{N_{\text{s}}\left(N_{\text{s}}-1\right)}\left[\overset{N_{\text{s}}-1}{\underset{n=1}{\sum }}\overset{N_{\text{s}}-n}{\underset{k=1}{\sum }}\delta \left(s\mp \frac{k}{N_{\text{s}}f_{\text{CR}}}\right)\right.\\ & & \left.+\overset{N_{\text{s}}-1}{\underset{n=1}{\sum }}\frac{n}{N_{\text{s}}}p\overset{N_{\text{s}}-1}{\underset{k=0}{\sum }}\overset{\infty }{\underset{l=1}{\sum }}{\left(1-p\right)}^{l-1}\delta \left(s\mp \frac{lN_{\text{s}}-n+k}{N_{\text{s}}f_{\text{CR}}}\right)\right] \end{array}$$

$$\begin{array}{ccc} & & +p\left(1-p\right)\overset{2N_{\text{s}}}{\underset{m=1}{\sum }}\overset{\infty }{\underset{l=0}{\sum }}{\left(1-p\right)}^{l}\frac{N_{\text{s}}-|m-N_{\text{s}}|}{N_{\text{s}}^{2}}\delta \left(s\mp \frac{m+lN_{\text{s}}}{N_{\text{s}}f_{\text{CR}}}\right). \end{array}$$ (14)

The terms in the square brackets describe the case where the postsynaptic neuron receives a stimulus in the same cycle as the presynaptic stimulus. The term in the last row describes the case where it does not receive a stimulus in the same cycle.

Next, we derive the correction terms $\delta p_{\text{intra/inter}}^{\text{bCR}}$ necessary to incorporate fast stimulation (1/*N*_s_ *τ* ≤ *f*_CR_ < 2/*N*_s_ *τ*) (Kromer et al., 2020). For such stimulation frequencies, exactly one stimulus is delivered before the delayed presynaptic spike arrives at the postsynaptic neuron. This results in a change of the sequence of time lags, considered for weight updates, relative to the case *f*_CR_ < 1/*N*_s_ *τ*. The difference in time lags will be incorporated in the correction functions $\delta p_{\text{intra/inter}}^{\text{bCR}}$ which have norm zero. The full distribution of time lags contributing to weight updates is then given by

$$p_{\text{intra/inter}}^{\text{bCR}}=\left\{\begin{array}{cc} p_{\text{intra/inter},0}^{\text{bCR}},\; & \text{for}\;f_{\text{CR}}<\frac{1}{N_{\text{s}}\tau }\\ p_{\text{intra/inter},0}^{\text{bCR}}+\delta p_{\text{intra/inter}}^{\text{bCR}},\; & \text{for}\;\frac{1}{N_{\text{s}}\tau }\leq f_{\text{CR}}<\frac{2}{N_{\text{s}}\tau }\\ 0,\; & \text{otherwise} \end{array}\right..$$ (15)

with $p_{\text{intra/inter}}^{\pm ,\text{bCR}}=p_{\text{intra/inter},0}^{\pm ,\text{bCR}}+\delta p_{\text{intra/inter}}^{\pm ,\text{bCR}}$ . In the present study, we do not consider the case $f_{\text{CR}}\geq \frac{2}{N_{\text{s}}\tau }$ and set $p_{\text{intra/inter}}^{\pm ,\text{bCR}}$ to zero for such parameter combinations.

First, we derive the correction term for $p_{\text{intra}}^{-,\text{bCR}}$ . For 1/*N*_s_ *τ* ≤ *f*_CR_ < 2/*N*_s_ *τ*, the sequence of time lags changes for all cases where the time lag between stimuli delivered to the post- and presynaptic neuron is 1/*N*_s_ *f*_CR_. This applies to the case where the presynaptic and postsynaptic neurons receive a stimulus at time (*N*_s_ − 1)/*N*_s_ *f*_CR_ of CR cycle *k* and the next stimulus at the beginning of CR cycle *k* + 1. The interstimulus intervals that lead to negative weight updates are now of length 1/*N*_s_ *f*_CR_ rather than of length 0, as for *f*_CR_ < 1/*N*_s_ *τ*. As this case occurs with probability $p/N_{\text{s}}^{2}$ , the corresponding correction function reads

$$\delta p_{\text{intra}}^{-,\text{bCR}}\left(s\right)=\frac{p}{N_{\text{s}}^{2}}\left(\delta \left(s-\frac{1}{N_{\text{s}f_{\text{CR}}}}\right)-\delta \left(s\right)\right).$$ (16)

We proceed similarly for $p_{\text{intra}}^{+,\text{bCR}}\left(s\right)$ and find the correction function

$$\delta p_{\text{intra}}^{+,\text{bCR}}\left(s\right)=\frac{p}{N_{\text{s}}^{2}}\left(-\delta \left(s-\frac{1}{N_{\text{s}f_{\text{CR}}}}\right)+\overset{\infty }{\underset{l=0}{\sum }}\frac{p{\left(1-p\right)}^{l}}{N_{\text{s}}}\overset{N_{\text{s}}-1}{\underset{k=0}{\sum }}\delta \left(s-\frac{N_{\text{s}}+1+k+lN_{\text{s}}}{N_{\text{s}}f_{\text{CR}}}\right)\right).$$ (17)

The correction functions for interpopulation synapses $\delta p_{\text{inter}}^{\pm ,\text{bCR}}\left(s\right)$ are longer expressions and are given in the Supplementary Material for the reader’s convenience.

The sign of $J_{\text{intra/inter}}^{\text{X}}$ determines whether synaptic weights are expected to weaken (-) or strengthen (+) during ongoing stimulation. Accordingly, we expect stimulation that weakens synapses sufficiently to drive the network into an attractor of a weakly connected stable desynchronized state, whereas we expect stimulation that strengthens synapses to drive the network into a strongly connected, synchronized state. Throughout the present paper, zeros of $J_{\text{intra/inter}}^{\text{X}}$ , Eq. (4), were used to approximate the boundary between stimulation parameters that lead to long-lasting desynchronization $\left(J_{\text{intra/inter}}^{\text{X}}<0\right)$ and parameters that lead to long-lasting synchronization $\left(J_{\text{intra/inter}}^{\text{X}}>0\right)$ .

## 3 Results

We study the consequence of randomized stimulus amplitudes on the acute and long-lasting effects of CR and tCR stimulation. To this end, we perform simulations of networks of 10^3^ LIF neurons with STDP (see Methods) and compare the results to our theoretical predictions (see Methods). Simulated networks were prepared in the synchronized state (see Methods). If not stated otherwise, stimulation was delivered for 1,000 s.

Motivated by therapeutic brain stimulation in the context of Parkinson’s disease, we focus on the effect of stimulation on neuronal synchrony. We distinguish between acute effects and long-lasting effects on synchronization (see Methods). Acute effects were quantified by the acute Kuramoto order parameter, ${\bar{\rho }}_{\text{ac}}$ , quantifying the degree of neuronal synchrony during stimulation. Long-lasting effects were quantified by the long-lasting Kuramoto order parameter, ${\bar{\rho }}_{\text{ll}}$ , quantifying the degree of neuronal synchrony 1,000 s after cessation of stimulation. Additionally, we analyze the mean synaptic weight shortly before the cessation of stimulation ⟨*w*⟩_ac_. ⟨*w*⟩_ac_ quantifies the effect of stimulation on the synaptic connectivity. In particular, stimulation led to an overall weakening of synaptic connections if ⟨*w*⟩_ac_ < *w*_0_ and to an overall strengthening of synaptic weights if ⟨*w*⟩_ac_ > *w*_0_. *w*_0_ ≈ 0.38 is the mean synaptic state in the stable synchronized state. In an earlier study, we found that ⟨*w*⟩_ac_ is highly predictive of the long-term outcome of stimulation. For ⟨*w*⟩_ac_⪅0.27, the network typically approached the stable desynchronized state, whereas it approached the synchronized state for larger ⟨*w*⟩_ac_ (see Figure 3 in Kromer et al. (2020)). Furthermore, the immediate after-effect on synchronization is quantified by the Kuramoto order parameter ${\bar{\rho }}_{\text{af}}$ , evaluated shortly after cessation of stimulation (see Methods for details). We are particularly interest in stimulation techniques that cause long-lasting desynchronization, i.e., desynchronization effects that persist after cessation of stimulation.

**FIGURE 3:**
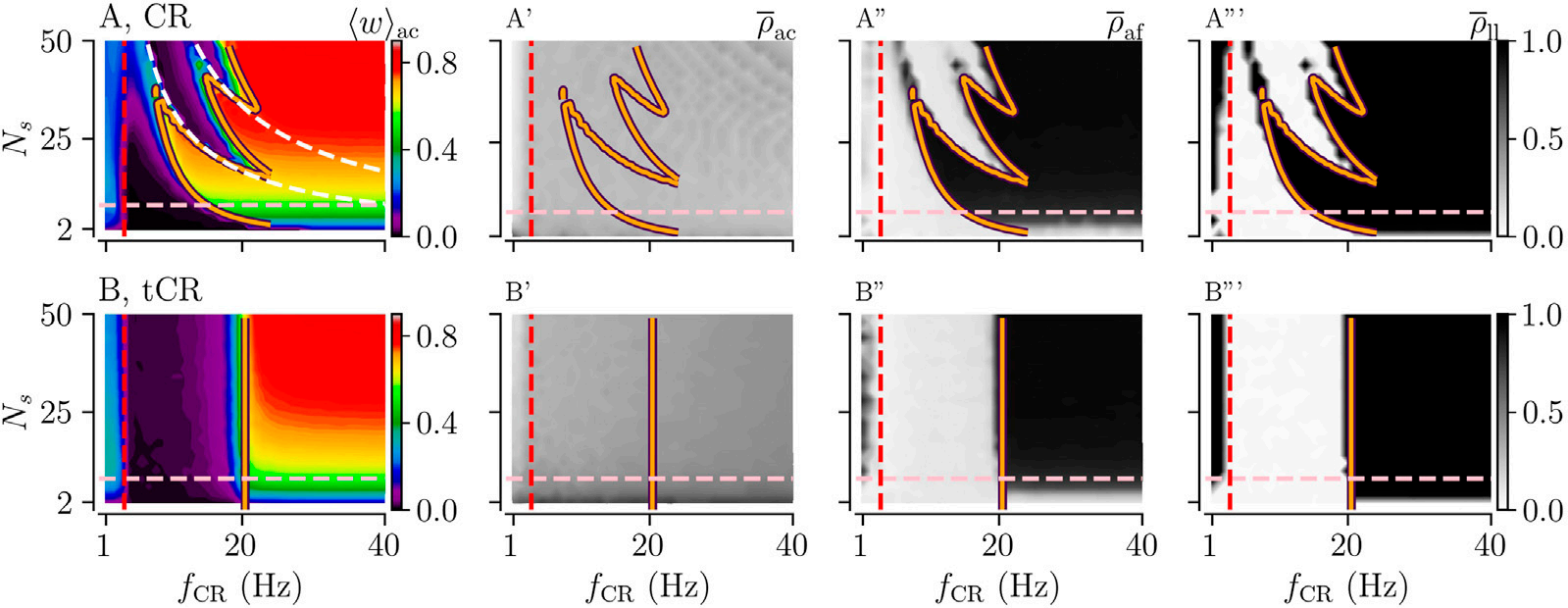
Acute and long-lasting effects of CR and tCR stimulation as a function of the stimulation frequency *f*_CR_ and the number of subpopulations *N*_*s*_. (A, B): The mean synaptic weight, ⟨*w*⟩_ac_, at the end of a 1,000 s stimulation duration for both CR (A) and tCR (B) stimulation. The orange curves show the zero-contour line of $J_{\text{inter}}$ , Eq. 4, taken from Kromer et al. (2020). The white dashed curves in panel A mark the curves 1/*N*_s_ *f*_CR_ = *τ* and 2/*N*_s_ *f*_CR_ = *τ*. The former one marks the limit of the zeroth order approximation used for the calculation of $J_{\text{inter}}^{\text{CR}}$ and the latter one the limit of the first correction term. The vertical red dashed line marks the frequency of the underlying synchronous rhythm (≈3.5 Hz). The pink horizontal line marks the region of clinically relevant values of *N*_s_⪅8, based on currently used DBS lead technology (Krauss et al., 2021). A′,B’: The acute after-effect on synchronization as quantified by the Kuramoto order parameter, ${\bar{\rho }}_{\text{af}}$ , time-averaged over a 10-s interval after cessation of the stimulation. A″,B”: The long-lasting desynchronization effects quantified by the Kuramoto order parameter, ${\bar{\rho }}_{\text{ll}}$ , averaged over a 10-s interval 1,000 s after cessation of stimulation. Here the stimulation duration was set to *T*_stim_ = 1,000 s and *A*_stim_ = 1. Note that results for CR were previously published in Kromer et al. (2020).

### 3.1 Temporally Uncorrelated Multi-Site CR Stimulation Improves Frequency-Robustness of Long-Lasting Desynchronization Effects

First, we considered the case of constant stimulus amplitudes, *A*_stim_ = 1. Results for CR and tCR stimulation are shown in Figure 3. Here, acute and long-lasting effects of either stimulation pattern on neuronal synchrony are shown as a function of the number of subpopulations, *N*_s_, and the stimulation frequency, *f*_CR_. For regular CR stimulation, the mean synaptic weight and neuronal synchrony after cessation of stimulation, i.e., ${\bar{\rho }}_{\text{af}}$ and ${\bar{\rho }}_{\text{ll}}$ , show a nonlinear dependence on *N*_s_ and *f*_CR_ (Figure 3A). This is in accordance with previous studies, which analyzed this phenomenon in more detail (Kromer et al., 2020). In contrast, corresponding measures show only a weak dependence on *N*_s_ for tCR stimulation. This indicates that no such nonlinearities occur for the tCR stimulation pattern (Figure 3B).

Long-lasting desynchronization $\left({\bar{\rho }}_{\text{ll}}\approx 0\right)$ by tCR stimulation does occur in a broad frequency range ( ≈3−20 Hz) (Figure 3B). The boundary of the parameter region in which stimulation entails long-lasting desynchronization is well-described by the theoretically predicted boundary of the parameter region in which stimulation strenghtens interpopulation synapses, i.e., synapses that connect neurons of different subpopulations (Figure 3A, B). This indicates that the transition between long-lasting desynchronization and long-lasting synchronization occurs because stimulation-induced synaptic potentiation of interpopulation synapses dominates over synaptic depression at high stimulation frequencies. The particular frequency value at which this transition occurs depends on the shape of the STDP function, Eq. 1. For low stimulation frequencies ( <4 Hz), long-lasting desynchronization effects of tCR stimulation are less pronounced than for CR stimulation. Furthermore, CR stimulation yields weaker acute synchronization than tCR stimulation throughout the *N*_s_-*f*_CR_ parameter plane (Figure 3A, B).

In the parameter region where our theoretical results predict a strengthening of interpopulation synapses (right of orange curve in Figure 3), we find a pronounced dependence of the mean synaptic weight on the number of separately stimulated subpopulations, *N*_*s*_. This is because *N*_*s*_ determines the fraction of interpopulation synapses to intrapopulation synapses. For small *N*_*s*_, a substantial portion of the synapse are intrapopulation synapses. Intrapopulation synapses are predicted to weaken during ongoing CR and tCR stimulation by our theory. In contrast, for large *N*_*s*_ most synapses are interpopulation synapses. Their dynamics depends on the stimulation parameters. For high stimulation frequencies (right of orange curve in Figure 3), only interpopulation synapses strengthen during ongoing stimulation which, eventually, leads to an *N*_*s*_-dependent mean synaptic weight given by the fraction of interpopulation synapses.

### 3.2 Uniformly Distributed Stimulus Amplitudes Improve Long-Lasting Desynchronization for Fast Stimulation

Next, we study how a randomization of stimulus amplitudes *A*_stim_ affects these results. To this end, we consider two randomization schemes: uniform randomization (u) and binary randomization (b). For uniform randomization, each subpopulation receives one stimulus with a uniformly distributed amplitude *A*_stim_ ∈ [0, 1] per CR cycle. Corresponding stimulation patterns are marked by the letter ‘u’, e.g., uCR and utCR. For binary randomization, each subpopulation receives a stimulus with amplitude *A*_stim_ = 1 with probability *p* during a CR cycle. Corresponding stimulation patterns are marked by the letter ‘b’, e.g., bCR and btCR.

**FIGURE 4:**
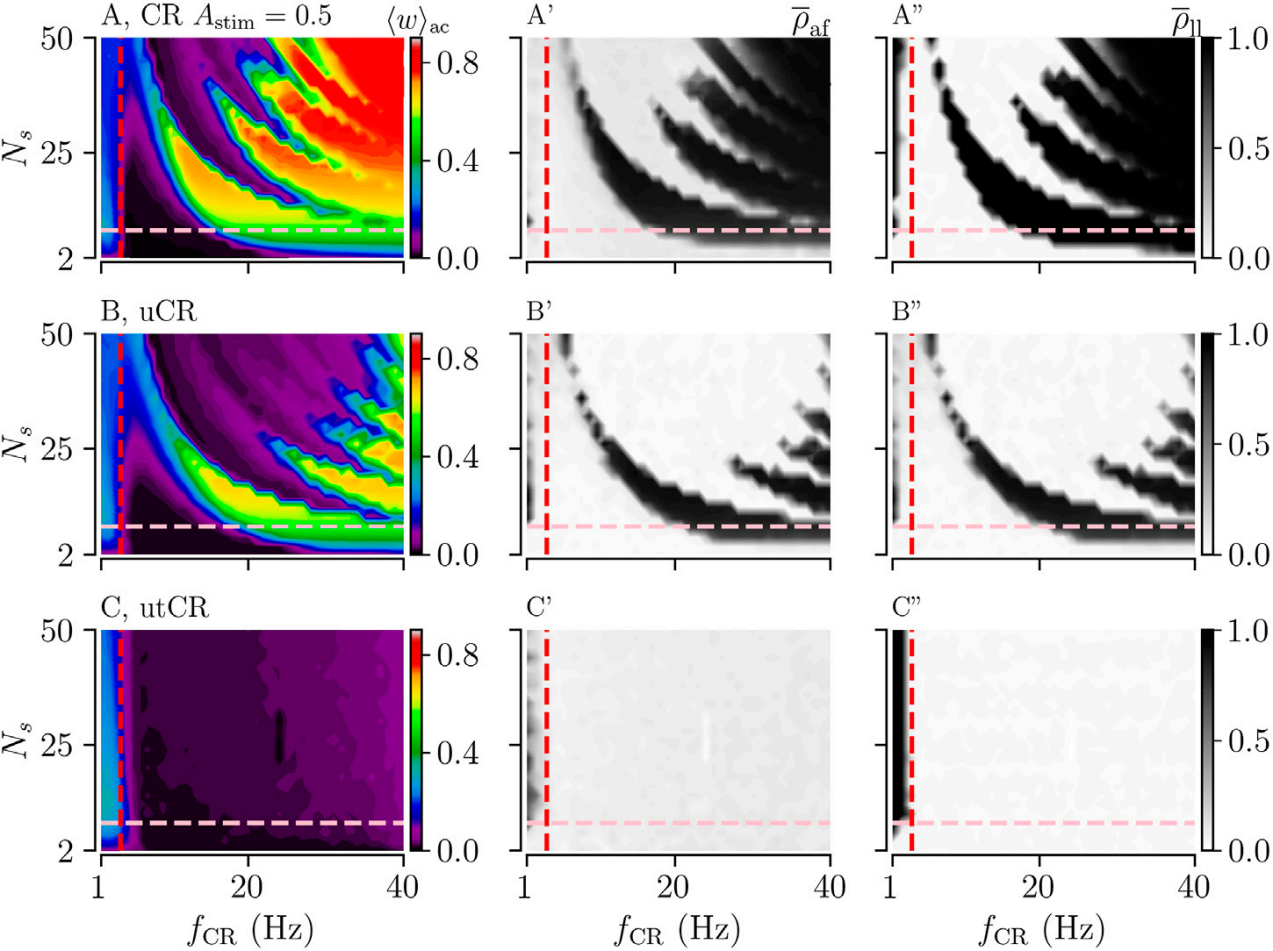
Acute and long-lasting effects of CR, uCR, and utCR stimulation with randomly distributed stimulus amplitudes (A,B,C): The acute mean synaptic weights, ⟨*w*⟩_ac_, at the end of the 1,000 s stimulation duration for CR (A), uCR (B), and utCR (C) stimulation. The vertical red dashed line marks the frequency of the underlying synchronous rhythm (≈3.5 Hz). The pink horizontal line marks the region of clinically relevant values of *N*_s_ (see caption of Figure 3). A′,B′,C′: The acute after-effect on synchronization as quantified by the Kuramoto order parameter, ${\bar{\rho }}_{\text{af}}$ , time-averaged over a 10-s interval after cessation of stimulation. A″,B″,C″: The long-lasting desynchronization effects quantified by the Kuramoto order parameter, ${\bar{\rho }}_{\text{ll}}$ , averaged over a 10-s interval 1,000 s after cessation of stimulation. The stimulation duration was set to *T*_stim_ = 1,000 s.

First, we consider the case of uniformly distributed stimulus amplitudes. Simulation results for uCR and utCR are shown in Figure 4. For both patterns, the overall mean stimulus amplitude ⟨*A*_stim_⟩ is 1/2. For comparison, we show results for CR with a constant stimulus amplitude *A*_stim_ = 1/2 in Figure 4A.

We find that long-lasting desynchronization occurs in a substantially bigger portion of the *N*_s_-*f*_CR_ parameter plane if stimulus amplitudes are uniformly randomized. For uCR stimulation, the nonlinear parameter dependence of long-lasting effects is still visible; however, it is less pronounced for intermediate stimulation frequencies (3–30 Hz). utCR stimulation entails long-lasting desynchronization in an even broader frequency range than tCR stimulation (compare Figures 3B-B‴ and Figures 4C-C”).

### 3.3 Random Stimulus Removal Improves Long-Lasting Desynchronization Effects of Fast Stimulation

Uniformly distributed stimulus amplitudes increase the parameter regions where CR and tCR stimulation entail long-lasting desynchronization (Figure 4). Next, we study whether this effect can be reproduced by randomly removing stimuli from the stimulus pattern. This is implemented by considering binarily distributed stimulus amplitudes, i.e., with probability *p* a subpopulation receives a stimulus of strength *A*_stim_ = 1 and with probability 1 − *p* no stimulus is delivered (*A*_stim_ = 0).

**FIGURE 5:**
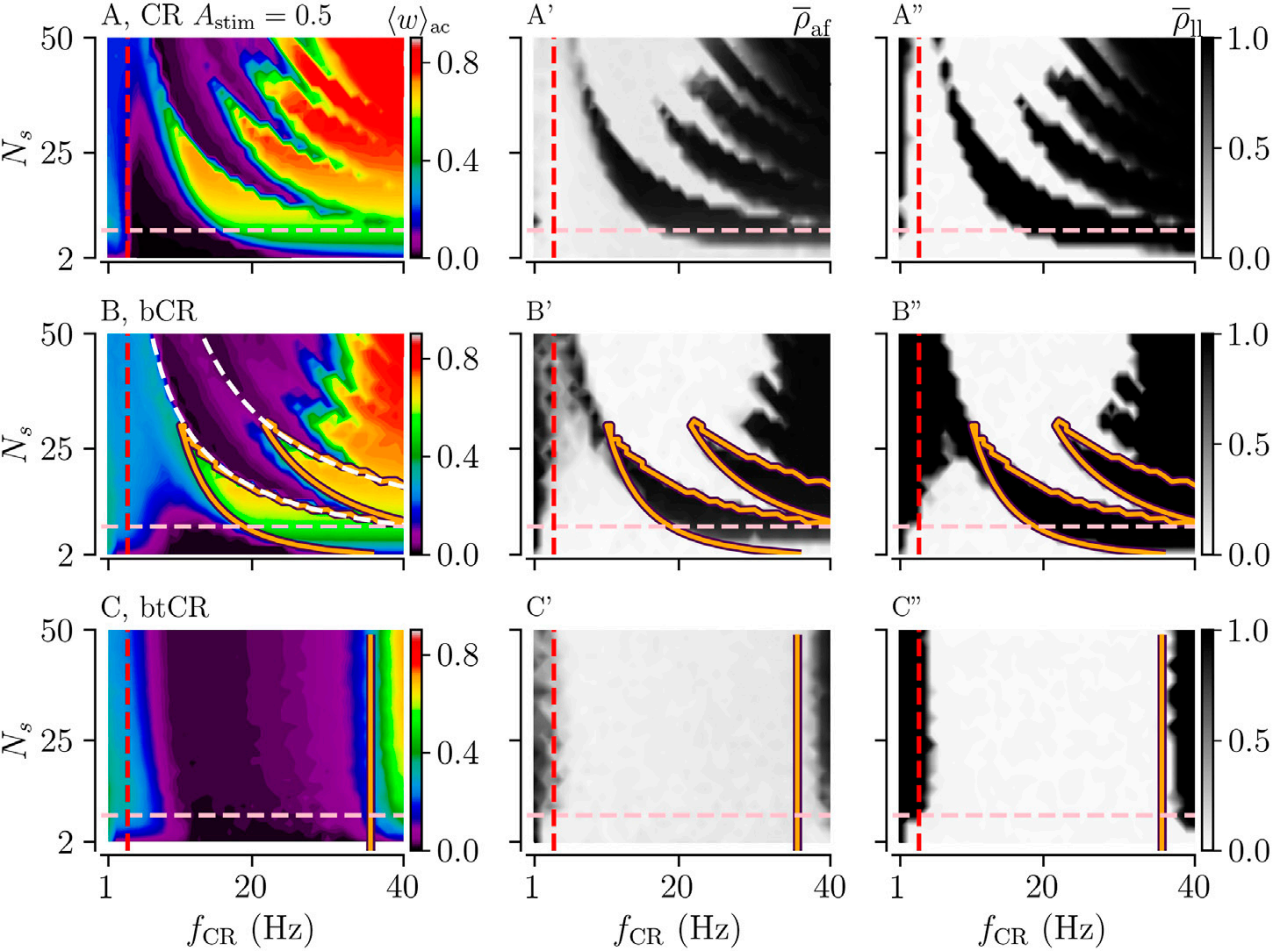
Effects of CR, bCR, and btCR stimulation on the mean synaptic weight and on synchronization after cessation of stimulation. The acute mean synaptic weight, ⟨*w*⟩_ac_, at the end of the 1,000 s stimulation duration is shown for CR (A), bCR (B), and btCR stimulation (C) in the first column. The orange curves show the zero-contour line of $J_{\text{inter}}^{\text{bCR}}$ as obtained from Eq. 4. The white dashed curves in panel B mark the curves 1/*N*_s_ *f*_CR_ = *τ* and 2/*N*_s_ *f*_CR_ = *τ*. The former one marks the limit of the zeroth order approximation used for the calculation of $J_{\text{inter}}^{\text{bCR}}$ and the latter one the limit of the first correction term. The vertical red dashed line marks the frequency of the targeted synchronous rhythm (≈3.5 Hz). The pink horizontal line marks the region of clinically relevant values of *N*_s_ (see caption of Figure 3). A′,B′,C’: The acute after-effect on synchronization as quantified by the Kuramoto order parameter, ${\bar{\rho }}_{\text{af}}$ , time-averaged over a 10-s interval immediately after cessation of stimulation. A″,B″,C″: Long-lasting desynchronization effects quantified by the Kuramoto order parameter, ${\bar{\rho }}_{\text{ll}}$ , averaged over a 10-s interval 1,000 s after cessation of stimulation. Parameters: Stimulation was delivered for 1,000 s, *A*_stim_ = 0.5 in A,A′, and A″.

Results for *p* = 1/2 are plotted in Figure 5. Panels **B–B”** show results for bCR simulation and panels **C–C”** results for btCR stimulation. For comparison, we show results for regular CR stimulation with constant stimulus amplitudes *A*_stim_ = 0.5 in panels **A–A”**. Binarily distributed stimulus amplitudes extend the parameter region in which bCR and btCR stimulation entail long-lasting desynchronization towards higher stimulation frequencies (Figures 5B-B” and **C-C”** as compared to Figures 5A-A” and Figure 3). However, bCR and btCR stimulation perform worse than stimulation with constant stimulus amplitudes for low stimulation frequencies (Figure 5).

Binarily distributed stimulus amplitudes have a qualitatively similar effect as uniformly distributed amplitudes with the same mean stimulus amplitudes ⟨*A*_stim_⟩ = 0.5; however, the frequency range in which long-lasting desynchronization can be achieved is smaller for binarily distributed stimulus amplitudes than for uniformly distributed stimulus amplitudes (compare Figure 4 and Figure 5).

**FIGURE 6:**
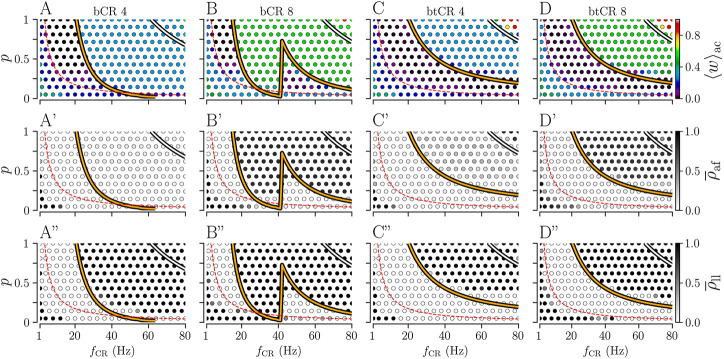
Effect of bCR and btCR stimulation as function of the stimulation frequency, *f*_CR_, and the number of stimulated subpopulations, *N*_*s*_. Columns show results for bCR stimulation with *N*_*s*_ = 4 (bCR 4) and with *N*_*s*_ = 8 (bCR 8), and for btCR stimulation with *N*_*s*_ = 4 (btCR 4) and with *N*_*s*_ = 8 (btCR 8). Rows show results for the acute mean synaptic weight, ⟨*w*⟩_ac_, evaluated at the end of the stimulation duration (top); the acute after-effect on synchronization as quantified by the Kuramoto order parameter, ${\bar{\rho }}_{\text{af}}$ , evaluated for a 10 s time interval immediately after cessation of stimulation (middle row); and the long-lasting effect on synchronization as quantified by the Kuramoto order parameter, ${\bar{\rho }}_{\text{ll}}$ , evaluated for a 10 s time interval 1,000 after cessation of stimulation. Colored thick curves show zero-contour lines of $J_{\text{inter}}^{\text{X}}$ (orange) and $J_{\text{intra}}^{\text{X}}$ (white) for respective stimulation patterns (X) obtained from Eq. 4. The red dashed curve represents parameter combinations for which the mean frequency of stimulus deliveries (*pf*_CR_) equals the frequency of collective spiking events in the synchronized state ( ≈3.5 Hz). Data points represent averages over three network realizations. Parameters: *A*_stim_ = 1.

Next, we analyze how the results depend on the fraction of delivered stimuli *p*. Results for bCR and btCR stimulation with fixed numbers of stimulation sites *N*_s_ = 4 and *N*_s_ = 8 are shown in Figure 6.

For bCR stimulation, long-lasting desynchronization effects are sensitive to *p* and the stimulation frequency. They are most robust with respect to changes of *p* for low stimulation frequencies ( <20 Hz) (Figures 6A,B). For higher stimulation frequencies, low *p* typically leads to more pronounced long-lasting desynchronization; however, for larger *N*_s_ we find an intermediate frequency range in which stimulation does not entail long-lasting desynchronization (Figure 6B).

Pronounced long-lasting synchronization occurs for high stimulation frequencies *f*_CR_ and large fractions of delivered stimuli *p*. Its boundary was well described by the zeros of $J_{\text{inter}}^{\text{bCR}}$ , Eq. 4. This suggests that strong interpopulation synapses are critical for synchronization. The mentioned intermediate frequency range for which bCR stimulation does not entail long-lasting desynchronization is well-reproduced by our theory. This suggests that this region results from a delay-induced effect, similar to the one leading to the patterned region of long-lasting synchronization in Figure 3A (see also Kromer et al. (2020)). In more detail, a sudden transition from $J_{\text{inter}}^{\text{bCR}}>0$ (strengthening of interpopulation synapses) to $J_{\text{inter}}^{\text{bCR}}<0$ (weakening of interpopulation synapses) occurs at 1/*N*_s_ *f*_CR_ = *τ*, i.e., when the presynaptic spike arrival occurs right when the next stimulus is delivered (with probability *p*). Another parameter region with long-lasting synchronization is found for low *f*_CR_ and low *p*. Here, stimulation is too slow to destabilize the initial synchronous state of the network.

Results for btCR stimulation are shown in Figures 6C-C” and D-D”. We find that the parameter region of long-lasting desynchronization is substantially bigger for btCR stimulation than for bCR stimulation. For higher stimulation frequencies, lower fractions of delivered stimuli *p* are required for long-lasting desynchronization. Fewer stimuli are delivered for lower *p*, resulting in fewer stimulus-triggered neuronal spikes. On average, this leads to longer time lags between post- and presynaptic spikes and reduces the contribution of synaptic LTD to the synaptic weight dynamics.

The zeros of $J_{\text{inter}}^{\text{btCR}}$ approximate the boundary of the parameter region with long-lasting synchronization well, occurring for high stimulation frequencies and high probabilities of stimulus delivery. We also find high values of the mean synaptic weight for very high *f*_CR_ and *p* (upper right corners of Figures 6C,D). There, stimulation strengthens inter- and intrapopulation synapses. This is also predicted by our theory as both $J_{\text{intra}}^{\text{btCR}}$ and $J_{\text{inter}}^{\text{btCR}}$ are positive in this parameter region. Remarkably, the shapes of the zero contour lines of $J_{\text{intra}}^{\text{btCR}}$ and $J_{\text{inter}}^{\text{btCR}}$ indicate that the parameter *p* and the stimulation frequency *f*_CR_ can be tuned to either weaken both intra- and interpopulation synapses (below the white and the orange curve in Figure 6), strengthen interpopulation synapses while weakening intrapopulation synapses (above the orange and below the white curve in Figure 6), or strengthen both intra- and interpopulation synapses (top right corner of Figure 6).

### 3.4 Less Stimulation Current Required for Uniformly Distributed Stimulus Amplitudes

So far, effects of all stimulus patterns were compared for the same stimulation duration. For clinical application, however, it is important to study long-lasting desynchronization effects as a function of the integral stimulation current, IC. The latter is strongly related to the battery lifetime of DBS pulse generators and the risk of unwanted side effects, for instance, due to current spread to neighboring tissue or due to too strong stimulation of the target region (see Krack et al. (2002) for a summary of possible side effects).

We calculate the overall integral stimulation current IC injected during a time interval *t* ∈ [0, *T*] as

$$\text{IC}\left(T\right)≔\overset{T}{\underset{0}{\int }}dt\;\underset{\begin{array}{c} i=0 \end{array}}{\sum }A_{\text{stim},i}\delta \left(t-t_{i}\right).$$ (18)

*A*_stim,*i*_ is the amplitude of the stimulus delivered at time *t*_*i*_ to a subpopulation. The sum runs over all realizations of stimulus amplitudes delivered to the *N*_s_ subpopulations. Thus, for the case of constant *A*_stim,*i*_ = 1 and *p* = 1, IC increases by *N*_s_ during individual CR cycles. Note that, in general, *A*_stim,*i*_ and IC are random variables. For a fixed time interval *T*, IC measures the total strength of administered stimuli. Large values of IC indicate that either strong stimuli were administered or rather weak stimuli were administered over a long time interval.

**FIGURE 7:**
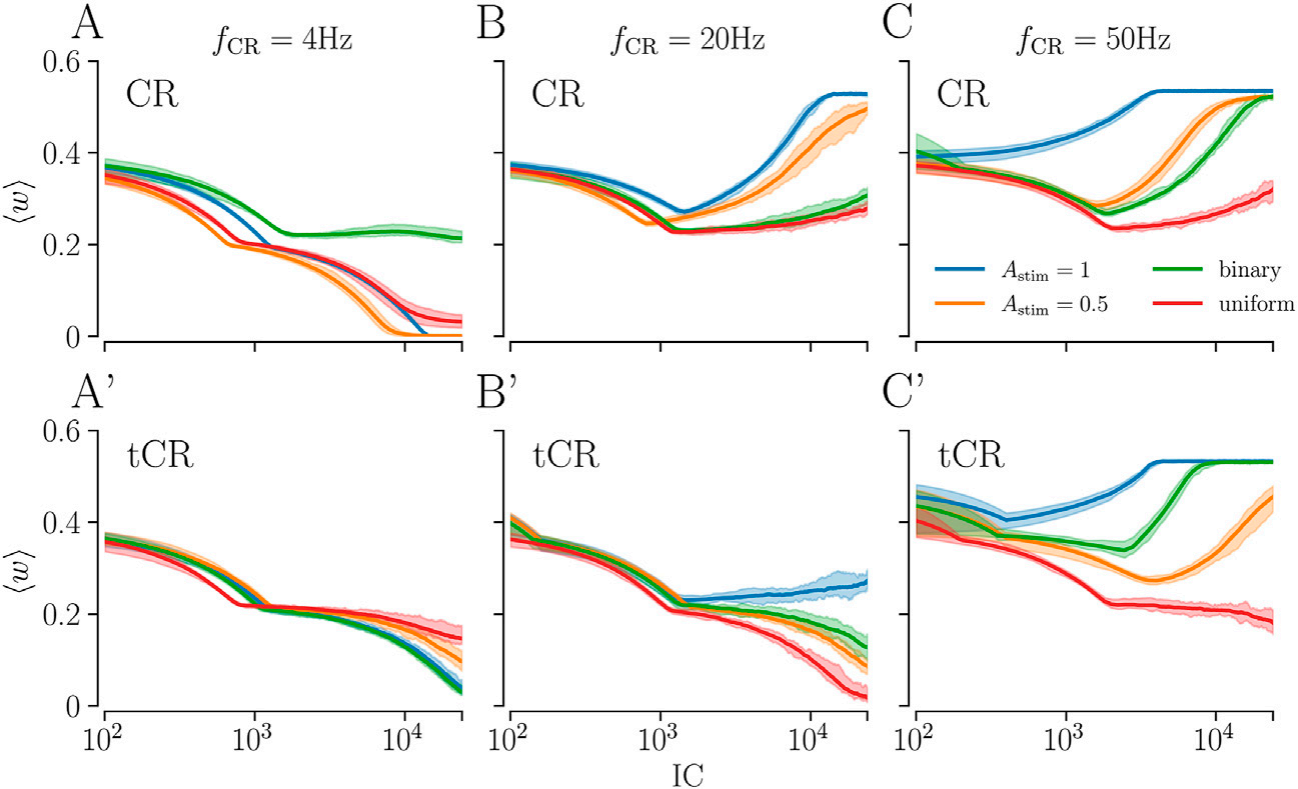
The mean synaptic weight during ongoing stimulation as a function of the integral stimulation current for different stimulation patterns. **(A–C)**: Results for CR with *A*_stim_ = 0.5, 1, bCR (green), and uCR stimulation (red) for slow **(A)**, intermediate **(B)**, and fast stimulation **(C)** (see labels). On the *x*-axis, we show the overall integral stimulation current (Eq. 18). Curves show averages over 12 network and stimulus pattern realizations and the shaded region corresponds to the range between the minimum and maximum across all network realizations. Panels A′-C′ show corresponding results for tCR, btCR (‘binary’, green curves), and utCR (‘uniform’, red curves) stimulation. Parameters: *N*_s_ = 8, *p* = 0.5 for bCR and btCR stimulation.

Figure 7 shows simulation results for different stimulation patterns for fixed *N*_s_ = 8 and different stimulation frequencies. Results for CR, uCR, and bCR stimulation are shown in Figures 7A–C. We find that these stimulation patterns perform best for low stimulation frequencies (*f*_CR_ = 4 Hz, Figure 7A). Here, all of these patterns lead to a substantial weakening of synapses. For higher stimulation frequencies, we typically find a reduction of the mean synaptic weight for small IC, whereas they strengthen for large IC (*f*_CR_ = 20, 50 Hz, Figures 7B,C). These two distinct regimes occur due to the different stimulation-induced dynamics of intra- and interpopulation synapses. For most stimulation patterns, $J_{\text{intra}}^{X}$ is negative, which leads to the weakening of intrapopulation synapses. Then, once the weights of these synapses approach zero, the dynamics of the mean synaptic weight is solely determined by that of interpopulation synapses. Thus, the weight dynamics for high IC yields information about the dynamics of interpopulation synapses. For IC → *∞* the mean synaptic weight approaches a stationary value given by the fraction of synapses that weaken. This ratio is closely related to the relative portion of inter- and intrapopulation synapses if stimulation strengthens one, while weakening the other type of synapses.

Results for tCR, btCR, and utCR stimulation are shown in Figures 7A′-C′. We find a qualitatively similar frequency dependence of the dynamics of the mean synaptic weight as for CR, bCR, and uCR stimulation. Remarkably, btCR and utCR stimulation outperform tCR stimulation with *A*_stim_ = 1 for intermediate stimulation frequencies. However, their performance is qualitatively similar to that of tCR stimulation with *A*_stim_ = 0.5 for *f*_stim_ = 20 Hz (Figure 7B’). For high stimulation frequencies, utCR stimulation yields the most pronounced weakening of synaptic weights (Figure 7C’).

Next, we consider the amount of stimulation current IC required for a substantial reduction of the mean synaptic weight. For intermediate and high stimulation frequencies, utCR stimulation typically leads to the lowest mean synaptic weight for a fixed value of IC (Figure 7). The only exemption is the low-IC region in Figure 7B, where CR stimulation with *A*_stim_ = 0.5 leads to a lower mean synaptic weight than utCR stimulation. For low stimulation frequencies, patterns with uniformly distributed stimulus amplitudes perform well for low IC, however they are outperformed by patterns with constant *A*_stim_ = 0.5 for high IC (Figures 7A,A′). Here, btCR and CR with *A*_stim_ = 1 outperform utCR and CR with *A*_stim_ = 0.5 (Figure 7A’).

Note, for regular CR (with *A*_stim_ = 0.5 or 1) as well as uCR, both delivered at *f*_CR_ = 4 Hz, we observe the most robust reduction of the mean synaptic weight with respect to large variations of IC (Figure 7A), compared to all other combinations of stimulation frequencies *f*_CR_ and stimulation patterns (Figures 7B,C,A′,B′,C′).

## 4 Discussion

In the present paper, we studied the effect of isolated stimulus amplitude randomization, isolated stimulus timing randomization as well as combinations thereof on the parameter robustness of long-lasting desynchronization effects of CR stimulation in networks of LIF neurons with STDP. Long-lasting desynchronization is observed when a sufficient weakening of synaptic connections occurs during the stimulation. Such weakening may drive the network into a stable desynchronized state, which allows desynchronized activity to persist after cessation of stimulation (Tass and Majtanik, 2006; Kromer et al., 2020). We considered regular CR and temporally uncorrelated CR stimulation, i.e., CR with stimulus timings that are randomized within CR cycles and uncorrelated between stimulus channels. In addition, two distributions of stimulus amplitudes were considered: a uniform distribution and a binary distribution. For binarily distributed stimulus amplitudes, a random fraction of stimuli was removed from the stimulus pattern (*A*_stim_ = 0), while the remaining stimuli had amplitude *A*_stim_ = 1. We find that randomization of stimulus amplitudes extends the parameter region of stimulation-induced long-lasting desynchronization effects towards high stimulation frequencies. However, it slightly reduces desynchronization effects for low stimulation frequencies.

First, we studied regular CR and temporally uncorrelated CR with constant stimulus amplitudes. We set *A*_stim_ = 1 which corresponds to a strong stimulation regime where stimuli trigger neuronal spikes (Kromer et al., 2020). The regular CR pattern was used in previous computational studies (Popovych and Tass, 2012; Zeitler and Tass, 2015), preclinical studies (Tass et al., 2009; Tass et al., 2012b; Wang et al., 2016), and clinical studies in Parkinson’s disease patients where it was either delivered using DBS electrodes (Adamchic et al., 2014) or using vibrotactile fingertip stimulation (Syrkin-Nikolau et al., 2018; Pfeifer et al., 2021). It was also delivered to suppress binge alcohol drinking in mice (Ho et al., 2021) and as a treatment for tinnitus (Tass et al., 2012a; Munjal et al., 2021). Temporally uncorrelated CR was introduced in an earlier computational study, where it was referred to as uncorrelated multichannel noisy stimulation (Zeitler and Tass, 2018). In networks of LIF neurons, both stimulation protocols yield pronounced long-lasting desynchronization effects for stimulation frequencies in the range from one to five times the frequency of the targeted synchronous rhythm. This frequency range is independent of the number stimulated subpopulations for temporally uncorrelated CR (Figure 3B). Contrastingly, it may shrink substantially for unfavorable numbers of subpopulations for regular CR (Figure 3A). This shrinking was first reported in Kromer et al. (2020) (note that shorter stimulation durations were used in Kromer et al. (2020)). In that study, the pronounced dependence on the number of subpopulations was explained as a delay-induced effect. More specifically, transitions between parameter regions with long-lasting desynchronization and parameter regions with long-lasting synchronization occurred when integer multiples of the minimal interstimulus interval, 1/*f*_CR_ *N*_s_, equaled the delay time, *τ* (white dashed curves in Figure 3A). Thus, stimulus-triggered spikes cannot arrive at their postsynaptic neurons before the next stimulus is delivered (Kromer et al., 2020). This led to a sudden transition from stimulation-induced LTP (the presynaptic spike arrives shortly before the postsynaptic neuron fires a spike triggered by a later stimulus) to stimulation-induced LTD (the presynaptic spike arrives shortly after the postsynaptic neuron fires a spike triggered by a later stimulus). Remarkably, this effect does not occur for temporally uncorrelated CR rendering its long-lasting desynchronization effects more robust with respect to parameter changes (Figure 3B). This is in line with results from previous studies suggesting that a reduction of temporal correlations between stimuli improves the frequency robustness of long-lasting desynchronization effects (Kromer and Tass, 2020; Khaledi-Nasab et al., 2021a; Khaledi-Nasab et al., 2021b). Specifically, we added temporal jitter and found that this smoothes out these sudden transitions and leads to overall stronger stimulation-induced LTD (see Figure 3 in Khaledi-Nasab et al. (2021b)). However, we find that temporally uncorrelated CR performs slightly worse than CR at low stimulation frequencies (lower than the frequency of the targeted synchronous rhythm). This is in line with the results of Zeitler and Tass (2018). Zeitler et al. considered a network of coupled Hodgkin-Huxley neurons with excitatory and inhibitory synaptic connections and adjusted the stimulation frequencies to that of the targeted synchronous rhythm. They reported on average weaker long-lasting effects for temporally uncorrelated CR than for the regular CR stimulation.

In the present paper, we considered random stimulus amplitudes. First, we distributed stimulus amplitudes uniformly between *A*_stim_ = 0 (no stimulus is delivered) and the maximum stimulus amplitude *A*_stim_ = 1. For *A*_stim_ = 1, the integral current over the excitatory part of a single stimulus is strong enough to drive the LIF neurons’ membrane potentials over the spiking threshold (Kromer et al., 2020). We find that this type of stimulus amplitude randomization substantially extends the frequency range for long-lasting desynchronization effects towards higher stimulation frequencies. For uCR stimulation, this mainly affects the parameter region of large *N*_s_ (Figure 4B). In contrast, if combined with temporal randomization, stimulation with uniformly distributed stimulus amplitudes induces long-lasting desynchronization effects for stimulation frequencies at least up to twelve times the frequency of the synchronous target rhythm and arbitrary values of *N*_s_ (see results for utCR stimulation in Figure 4). Long-lasting desynchronization can be achieved for all considered numbers of subpopulations. For regular CR, we find that stimulus amplitude randomization reduces the delay-induced effects mentioned above. This substantially extends the parameter region in which long-lasting desynchronization can be achieved. However, this occurs mainly in the range of large numbers of subpopulations (Figure 4B).

To put our results for larger numbers of subpopulations *N*_s_ in perspective, we note that the clinical proof of concept data obtained with CR stimulation delivered to the STN in Parkinson’s patients were obtained with only two or three active circular stimulation contacts being located in the STN (Adamchic et al., 2014; Ebert et al., 2014). In general, in clinical applications, the number of subpopulations *N*_s_ is limited by the electrode design, the number and dimension of stimulation contacts and the size of the target brain region (Krauss et al., 2021). While recent multisite stimulation electrodes possess large numbers of stimulation contacts (Steigerwald et al., 2019), e.g., up to 32 (Contarino et al., 2014), it remains to be shown whether such electrodes can be used to deliver stimuli to large numbers of separate neuronal subpopulations in target brain areas, such as the STN, independently. Larger numbers of smaller, e.g., segmented rather than circular, stimulation contacts might enable more focal stimulation by selecting groups of active stimulation contacts, hence, providing more favorable outcome. However, larger numbers of stimulation contacts do not imply a larger number of subpopulations *N*_s_, since, due to the non-homogeneous anatomy of DBS targets, activation of particular subsets of stimulation contacts typically does not yield any therapeutic effects or even cause side effects. Further studies are required to understand the anatomy and physiology of the spatial dimensions of DBS target subpopulations to infer realistic ranges and, specifically, maximum values of *N*_s_. With these caveats in mind, in Figures 4, 5 we used the range *N*_s_⪅8 (pink dashed line) (Krauss et al., 2021) to illustrate a plausible clinically relevant range of *N*_s_ achievable with recent multi-contact DBS electrodes. In that range, however, uniform stimulus amplitude randomization alone does not improve the frequency robustness of long-lasting desynchronization effects compared to regular CR stimulation (Figures 4A,B,A”,B”) while binary stimulus amplitude randomization even worsens the frequency robustness of long-lasting desynchronization effects compared to regular CR (Figures 5A,B,A”,B”). By the same token, the advantage of stimulus timing randomization alone is rather limited (Figures 3A,B,A‴,B‴). However, double-random CR, i.e., CR with the combination of (uniform or binary) amplitude randomization as well as stimulus timing randomization, leads to more favorable long-term desynchronization for values of the stimulation frequency *f*_CR_ that exceed the intrinsic frequency of the abnormally synchronized target rhythm by a factor of five or more (Figures 4A,C,A”,C” as well as Figures 5A,C,A”,C”). If future clinical studies showed that a large number of neuronal subpopulations can be stimulated independently in related brain areas, double-random CR stimulation might induce more favorable long-lasting desynchronization effects, specifically for larger numbers of subpopulations *N*_s_.

Since weak stimuli may not trigger neuronal spikes, stimulus amplitude randomization may lead to more sparse stimulation-induced spike patterns than stimulation with constant amplitudes. Intuitively, this may lead to more long time lags between arrivals of stimulus-triggered presynaptic spikes and stimulus-triggered postsynaptic spikes, which may favor LTD for STDP rules where LTD dominates over LTP for long time lags (*τ*_+_ < *τ*_−_ and *β* > 1 in Eq. 1). Previous studies analyzed the firing rate dependence of the synaptic weight dynamics in neuronal networks with STDP. They found that lower firing rates typically result in weaker synaptic weights for asymmetric STDP functions with strong LTP for short positive and LTD for negative time lags (Equation 1) (Sjöström et al., 2001; Burkitt et al., 2004). A sparsening of the stimulation-induced spike pattern might therefore reduce synaptic potentiation. This may explain the observed extension of the frequency range for long-lasting desynchronization towards higher stimulation frequencies. To test this hypothesis, we considered a second type of stimulus randomization where only a random fraction *p* of stimuli was delivered: binary stimulus randomization. We find that binary stimulus randomization leads to qualitatively similar effects as uniform stimulus randomization (Figures 4, 5). This supports the hypothesis that the observed improvement of long-lasting effects at high stimulation frequencies results from the effective sparsening of the stimulation-induced spike pattern.

For the same mean stimulus amplitude, we find that stimulation with uniform stimulus amplitude randomization performs better for high stimulation frequencies than stimulation with binary stimulus amplitude randomization. We suggest that this arises from differences in the statistics of time lags between stimulation-induced post- and presynaptic spikes. These time lags determine synaptic weight updates via the STDP function (Eq. 1). In the present paper, we considered an asymmetric STDP function, *τ*_R_ > 1, for which synaptic LTP dominates for time lags that are short compared to the STDP decay time *τ*_+_ (Eq. 1). Synaptic LTD dominates for long time lags. For uniform stimulus amplitude randomization, a large portion of stimuli only triggers spikes when the LIF neurons’ membrane potentials are close to the spiking threshold, i.e., after it left the refractory period following its previous spike. Such stimuli typically lead to rather large interspike intervals. In contrast, for binary randomization, spikes are triggered independently of the neurons’ membrane potential. Thus, for high stimulation frequencies, binary randomization results in more short interspike intervals. We therefore expect more short time lags between post- and presynaptic spikes during stimulation with binary stimulus amplitude randomization than during stimulation with uniform stimulus amplitude randomization, which would then translate into more pronounced stimulation-induced synaptic LTP and less pronounced LTD (compare Figures 4B-B”,C-C”, and Figures 5B-B”,C-C”).

We derived theoretical approximations for the mean rate of weight change during CR and temporally uncorrelated CR with binary distributed stimulus amplitudes. Our theoretical results extend earlier results from Kromer and Tass (2020), Kromer et al. (2020), and Khaledi-Nasab et al. (2021b) to the case of temporally uncorrelated CR stimulation, and, for the first time, incorporate stimulation with binary stimulus amplitude randomization. Our theoretical approach builds on the assumption that each stimulus triggers a neuronal spike, and each spike is triggered by a stimulus. Thus, neuronal spiking due to the intrinsic dynamics, noise, and synaptic input is neglected. Previous studies found that corresponding results well approximated the synaptic weight dynamics during ongoing strong (*A*_stim_ ≈ 1) and fast stimulation, i.e., when the stimulation frequency is higher than that of the synchronous target rhythm.

Our theory predicts that the synaptic weight dynamics during ongoing binary randomized stimulation depends on the statistics of stimulus trains delivered to the postsynaptic and the presynaptic neuron. We distinguish between intrapopulation synapses, where both neurons belong to the same subpopulation, and interpopulation synapses, where both neurons belong to different subpopulations. For intrapopulation synapses, the post- and presynaptic neuron receive stimuli simultaneously. We find that these synapses typically weaken. In contrast, for interpopulation synapses, the post- and presynaptic neuron typically receive stimuli at different times. Our theory predicts that interpopulation synapses strengthen in a large part of the (*p*, *f*_CR_)-parameter space (Figure 6). We find that our simulation results are well-approximated by the zeros of the predicted mean rate of weight change of interpopulation synapses. In particular, long-lasting synchronization is observed when interpopulation synapses strengthen during stimulation. In contrast, long-lasting desynchronization occurs when interpopulation synapses weaken during stimulation (Figure 6). We find that deviations between theoretical predictions and simulations mostly occur for low values of *p*. Thus, when a significant portion of stimuli is removed from the stimulus pattern and the probability for long interstimulus intervals increases (Figure 6).

Our analysis of the CR patterns with binary stimulus amplitude randomization also yields information on the impact of stimulation parameters on long-lasting desynchronization effects. In particular, we studied the impact of the fraction of removed stimuli *p* and the stimulation frequency for fixed numbers of subpopulations. For temporally uncorrelated CR, we find that the higher the stimulation frequency, the more stimuli have to be removed to ensure long-lasting desynchronization (Figure 6C and D). In contrast, for regular CR stimulation with binary stimulus amplitude randomization, there is an intermediate frequency range where no long-lasting desynchronization is possible for certain unfavorable numbers of subpopulations (compare Figures 6A”,B”). This effect has a similar origin as the delay-induced effect described above. It occurs for stimulation frequencies where the presynaptic spikes arrive shortly before the next stimulus is delivered (Figure 5 and Kromer et al. (2020)).

Besides supporting long-lasting desynchronization for high stimulation frequencies, stimulus amplitude randomization also reduces the delivered stimulation current. This may be advantageous for possible clinical applications, as the integral modulus of the stimulation current is strongly related to the risk of unwanted side effects (Krack et al., 2002). We compared the stimulation-induced mean synaptic weight for the different stimulation patterns as a function of the integral stimulation current. We find that uniform randomization performs best for intermediate and high stimulation frequencies. The best results were obtained for double-random CR, i.e., temporally uncorrelated CR with uniform stimulus amplitude randomization (Figure 7). This indicates that uniform stimulus amplitude randomization does perform better for a fixed stimulation duration, and requires less integral stimulation current. This suggests that temporally uncorrelated CR with uniform stimulus amplitude randomization may be superior to other stimulation patterns if the stimulation frequency is substantially higher than that of the synchronous target rhythm. Using a basal ganglia model without synaptic plasticity, an earlier computational study analyzed the acute effects of CR stimulation with noise stimuli instead of charge-balanced pulses with random amplitudes (Liu et al., 2022). The authors found that such noisy stimulation is also efficient in inducing acute desynchronization. Here, we solely focused on long-lasting effects.

To the best of our knowledge, the present paper presents the first study that analyzes long-lasting desynchronization effects of stimulation patterns with randomized stimulus amplitudes. Some experimental studies considered DBS with temporally randomized stimulus patterns. However, these studies analyzed the acute effect of temporally randomized DBS and provided mixed results: Dorval et al. (2010) and Birdno et al. (2012) reported that temporally randomized DBS was inefficient in providing symptom alleviation (Dorval et al., 2010; Birdno et al., 2012), whereas Brocker et al. (2013) reported that irregular DBS led to improved performance of Parkinson’s disease patients in a fingertip task. It is currently not known, whether DBS with randomized stimulus amplitudes is safe and feasible.

Our promising results suggest that temporally randomized CR with uniform stimulus amplitude randomization might be suitable for desynchronizing brain rhythms across a wide frequency range. This might be advantageous in PD, where different symptoms are associated with abnormal synchrony in different frequency bands (Brown, 2003; Kühn et al., 2006; Weinberger et al., 2006; Steigerwald et al., 2008). Assuming that the interaction between rhythms is weak, the stimulation frequency could be adjusted to the fastest rhythm. Our computational results then suggest that such stimulation might also induce long-lasting desynchronization in subnetworks with pathological synchrony in lower frequency bands.

In the present study, we used a simple model of 10^3^ excitatory LIF neurons with STDP. Its low computational costs enabled us to perform detailed scans of the parameter space (Figures 3–6). In future studies, we anticipate working with more detailed neuron models specifically fit to target brain areas for HF DBS in PD, such as the basal ganglia (Terman et al., 2002; Fountas and Shanahan, 2017). Note that the more detailed conductance-based STN neuron model presented in (Terman et al., 2002) fired one spike per DBS pulse during HF DBS, similar to our LIF model (Rubin and Terman, 2004; Dorval et al., 2010). We anticipate studying the effect of stimulation in systems with multiple synchronous rhythms that interact. An interesting question for future studies would also be to which extend non-neural cells such as glia cells contribute to the therapeutic effects of stimulation. Growing evidence suggests that such cells play an important role in both PD pathogenesis (Booth et al., 2017) and the therapeutic mechanism of HF DBS (Vedam-Mai et al., 2012). We also aim at including physiological input from other brain areas. In a network model with additional, e.g., sensory input, after CR stimulation the synaptic connectivity pattern converged to a physiological connectivity pattern (Hauptmann and Tass, 2010). Given the complexity of such high-dimensional models with large numbers of parameters, predictions generated in simple models, e.g., the LIF model, may guide and inform the analysis of high-dimensional models, in a similar way as they have enabled the pre-clinical development (animal testing; (Tass et al., 2012b; Wang et al., 2016)) and clinical development (Adamchic et al., 2014) so far. Furthermore, we hope that our promising results inspire future studies using detailed computational models of target brain regions, e.g., for DBS in PD and other brain disorders, and preclinical and clinical studies on long-lasting therapeutic effects of randomized brain stimulation.

## Data Availability

The original contributions presented in the study are included in the article/Supplementary Material, further inquiries can be directed to the corresponding author.
