# Supplementary material for: Long-Lasting Desynchronization of Plastic Neuronal Networks by Double-Random Coordinated Reset Stimulation
Source: Front Netw Physiol. 2022 Apr 19;2:864859. doi: 10.3389/fnetp.2022.864859 (PMC10013062; doi:10.3389/fnetp.2022.864859)
Supplement: Supplementary file 1 [file DataSheet1.pdf]

Supplementary Material

1 NEURONAL NETWORK MODEL

In our LIF model spikes occur when the membrane potential v_i crosses a dynamic threshold potential v_i^{th}. Here, i is the index of the LIF neuron. The dynamics of v_i and v_i^{th} are given by Kromer and Tass (2020)

$$C_i \frac{\mathrm{d}v_i}{\mathrm{d}t} = g_{\text{leak}}(v_{\text{rest}} - v_i) + I_i^{\text{syn}}(t) + I_i^{\text{stim}}(t) + I_i^{\text{noise}}(t). \tag{S1}$$
$$\tau_{\text{th}} \frac{\mathrm{d}v_i^{\text{th}}}{\mathrm{d}t} = (v_{\text{rest}}^{\text{th}} - v_i^{\text{th}}).$$

$I_i^{\text{syn}}(t)$ is the synaptic input current, $I_i^{\text{stim}}(t)$ the stimulation current, and $I_i^{\text{noise}}(t)$ the noisy input current. C_i is the membrane capacitance, τ_{th} is the threshold time constant, $v_{\text{rest}}^{\text{th}}$ is the stationary threshold potential, v_{rest} the resting potential, and g_{leak} is the leak conductance.

Spike times are recorded at each threshold crossing. After a spike, the membrane potential is set to v_{spike} for a time period of t_{spike}. Then, an instantaneous reset is performed by setting $v_i \to v_{\text{reset}}$ and $v_i^{\text{th}} \to v_{\text{spike}}^{\text{th}}$.

We use the same parameters as Kromer et al. (2020), Khaledi-Nasab et al. (2021b), and Khaledi-Nasab et al. (2021a). Specifically, we use $v_{\text{rest}} = -38$ mV, $\tau_{\text{th}} = 5$ ms, $v_{\text{rest}}^{\text{th}} = -40$ mV, $t_{\text{spike}} = 1$ ms, $v_{\text{spike}} = 20$ mV, $V_{\text{reset}} = -67$ mV, and $g_{\text{leak}} = 0.02$ mS/cm^2. The membrane capacitances C_i are random variables and are drawn from a normal distribution with mean value of $\langle C_i \rangle = 3$ μF/cm^2 and standard deviation of $0.05\langle C_i \rangle$. These parameters are chosen such that the frequency and amplitude of membrane potential oscillations of isolated LIF neurons resembles those of oscillatory neurons in the rat STN (Bevan and Wilson, 1999).

Each neuron i receives noisy input, I_i^{noise}, which is obtained by feeding presynaptic Poisson spike trains with firing rate $f_{\text{noise}} = 20$ Hz into excitatory synapses (Ebert et al., 2014)

$$I_i^{\text{noise}} = g_i^{\text{noise}}(v_{\text{syn}} - v_i), \tag{S2}$$
$$\tau_{\text{syn}} \frac{\mathrm{d}g_i^{\text{noise}}}{\mathrm{d}t} = -g_i^{\text{noise}} + D \sum_{k_i} \delta(t_{k_i}^i - t).$$

The noise intensity is controlled by the parameter $D = 0.026$ mS/cm^2 scaling the strength of the Poisson input. $v_{\text{syn}} = 0$ mV is the synaptic reversal potential, $\tau_{\text{syn}} = 1$ ms the synaptic time scale, and $g_i^{\text{noise}}(t)$ the synaptic conductance.

$I_i^{\text{syn}}(t)$ is the excitatory synaptic input to neuron i, and it is given by

$$I_i^{\text{syn}} = g_i^{\text{syn}}(v_{\text{syn}} - v_i), \tag{S3}$$
$$\tau_{\text{syn}} \frac{\mathrm{d}g_i^{\text{syn}}}{\mathrm{d}t} = -g_i^{\text{syn}} + \frac{\kappa}{N} \sum_{j \in G_i} w_{ji} \sum_{lj} \delta(t - t_{lj}^j - t_{\text{d}}),$$

where $\kappa = 8$ mS/cm^2 is the coupling strength and g_i^{syn} is the synaptic conductance, $w_{ji} \in [0, 1]$ is the weight of the synapses between presynaptic neuron j and postsynaptic neuron i. The first sum runs over

all presynaptic neurons j of neuron i, and the second sum runs over the spikes of neuron j. We consider homogeneous synaptic delays of $t_{\mathrm{d}} = 3$ ms.

2 CORRECTION TERMS BCR STIMULATION

We give results for the correction terms for the mean rate of weight change of interpopulation synapses during bCR stimulation. These were derived as described in the main text. For $\delta p_{\text{inter}}^{-,\text{bCR}}(s)$, we find

$$\begin{aligned}\delta p_{\text{inter}}^{-,\text{bCR}}(s) &= \frac{p}{N_{\mathrm{s}}(N_{\mathrm{s}}-1)}\left[(N_{\mathrm{s}}-1)\delta\left(s-\frac{1}{N_{\mathrm{s}}f_{\mathrm{CR}}}\right)-\sum_{\xi=0}^{N_{\mathrm{s}}-2}\sum_{l=1}^{\infty}\sum_{k=0}^{N_{\mathrm{s}}-1}\frac{p(1-p)^{l-1}}{N_{\mathrm{s}}}\delta\left(s+\frac{lN_{\mathrm{s}}+k+\xi}{N_{\mathrm{s}}f_{\mathrm{CR}}}\right)\right]\\ &+\frac{p}{N_{\mathrm{s}}^2}\left[\delta\left(s-\frac{1}{N_{\mathrm{s}}f_{\mathrm{CR}}}\right)-\left(\frac{p}{N_{\mathrm{s}}-1}\sum_{k=1}^{N_{\mathrm{s}}-1}\delta\left(s+\frac{k}{N_{\mathrm{s}}f_{\mathrm{CR}}}\right)+\sum_{l=1}^{\infty}\frac{p(1-p)^{l}}{N_{\mathrm{s}}}\sum_{k=0}^{N_{\mathrm{s}}-1}\delta\left(s+\frac{lN_{\mathrm{s}}+k}{N_{\mathrm{s}}f_{\mathrm{CR}}}\right)\right)\right].\end{aligned} \tag{S4}$$

For $\delta p_{\text{inter}}^{+,\text{bCR}}(s)$, we find

$$\begin{aligned}\delta p_{\text{inter}}^{+,\text{bCR}}(s) &= \frac{p}{N_{\mathrm{s}}(N_{\mathrm{s}}-1)}\left[\sum_{\xi=0}^{N_{\mathrm{s}}-2}\sum_{l=1}^{\infty}\sum_{k=0}^{N_{\mathrm{s}}-1}\frac{p(1-p)^{l-1}}{N_{\mathrm{s}}}\delta\left(s-\frac{lN_{\mathrm{s}}+k-\xi}{N_{\mathrm{s}}f_{\mathrm{CR}}}\right)-(N_{\mathrm{s}}-1)\delta\left(s-\frac{1}{N_{\mathrm{s}}f_{\mathrm{CR}}}\right)\right]\\ &+\frac{p}{N_{\mathrm{s}}^2}\left[\sum_{l=1}^{\infty}\frac{p(1-p)^{l-1}}{N_{\mathrm{s}}}\sum_{k=0}^{N_{\mathrm{s}}-1}\delta\left(s-\frac{lN_{\mathrm{s}}+k+1}{N_{\mathrm{s}}f_{\mathrm{CR}}}\right)-\delta\left(s-\frac{1}{N_{\mathrm{s}}f_{\mathrm{CR}}}\right)\right].\end{aligned} \tag{S5}$$

REFERENCES

Bevan, M. D. and Wilson, C. J. (1999). Mechanisms underlying spontaneous oscillation and rhythmic firing in rat subthalamic neurons. *J. Neurosci.* 19, 7617–7628

Ebert, M., Hauptmann, C., and Tass, P. A. (2014). Coordinated reset stimulation in a large-scale model of the stn-gpe circuit. *Front. Comput. Neurosci.* 8, 154. doi:10.3389/fncom.2014.00154

Khaledi-Nasab, A., Kromer, J., and Tass, P. A. (2021a). Long-lasting desynchronization of plastic neural networks by random reset stimulation. *Front. Physiol.* 11, 622620. doi:10.3389/fphys.2020.622620

Khaledi-Nasab, A., Kromer, J. A., and Tass, P. A. (2021b). Long-lasting desynchronization effects of coordinated reset stimulation improved by random jitters. *Front. Physiol.* 12, 719680. doi:10.3389/fphys.2021.719680

Kromer, J. A., Khaledi-Nasab, A., and Tass, P. A. (2020). Impact of number of stimulation sites on long-lasting desynchronization effects of coordinated reset stimulation. *Chaos* 30, 083134. doi:10.1063/5.0015196

Kromer, J. A. and Tass, P. A. (2020). Long-lasting desynchronization by decoupling stimulation. *Phys. Rev. Res.* 2, 033101. doi:10.1103/PhysRevResearch.2.033101
